# Mitochondrial Dysfunction Induced by a Novel Salicylate‐Based 1,2,3‐Triazole Salt With Potent Antileishmanial Activity

**DOI:** 10.1111/cbdd.70227

**Published:** 2025-12-22

**Authors:** Bruno A. De Oliveira, Ari Sérgio de Oliveira Lemos, João Pedro Reis Costa Bastos, Luciana Maria Ribeiro Antinarelli, Ana Luiza Ribeiro de Freitas, Raissa Guedes Mattosinhos Ribeiro, Adolfo Firmino da Silva Neto, Elaine Soares Coimbra, Adilson David Da Silva

**Affiliations:** ^1^ Department of Chemistry, Institute of Exact Sciences Federal University of Juiz de Fora Juiz de Fora Minas Gerais Brazil; ^2^ Department of Parasitology, Microbiology and Immunology, Institute of Biological Sciences Federal University of Juiz de Fora Juiz de Fora Minas Gerais Brazil; ^3^ Department of Veterinary Medicine, Faculty of Medicine Federal University of Juiz de Fora Juiz de Fora Minas Gerais Brazil

**Keywords:** 1,2,3‐triazole derivatives, antileishmanial activity, *Leishmania amazonensis*, mitochondrial dysfunction, oxidative stress, salicylic ester derivatives

## Abstract

Leishmaniasis is a neglected disease caused by *Leishmania* spp., with limited treatment options and no available vaccine. In this study, novel 1,2,3‐triazole derivatives 4‐substituted with salicylic esters and their salts were synthesized and evaluated against *L. amazonensis*. While neutral compounds were inactive, their salts effectively inhibited both promastigote and intracellular amastigote growth. Compound **7** exhibited the highest antileishmanial activity and selectivity (IC_50_ against amastigotes of 6.23 ± 0.35 μM; SI of 17.81), surpassing miltefosine, the positive control. Additionally, this compound exhibited no hemolytic effect. Studies further demonstrated that at early time points (6 h), treatment with compound **7** led to a marked increase in total ROS and mitochondrial superoxide levels, although no change in mitochondrial membrane potential was detected, indicating an initial oxidative imbalance without immediate effects on ΔΨm. However, after 24 h of treatment, this sustained oxidative stress resulted in pronounced mitochondrial membrane potential hyperpolarization, while elevated ROS levels persisted. Throughout these assays, plasma membrane integrity remained unaffected in treated *L. amazonensis* promastigotes. In silico analyses suggested favorable pharmacokinetic properties and oral bioavailability. Collectively, these findings highlight compound **7** as a promising candidate for further studies, including in vivo assays using murine models of leishmaniasis.

## Graphical abstract

A novel salicylate‐based 1,2,3‐triazole salt was synthesized and demonstrated potent and selective activity against *Leishmania amazonensis*. The most active compound induced mitochondrial dysfunction without hemolytic effects. In silico analyses also suggested favorable pharmacokinetic properties and oral bioavailability.

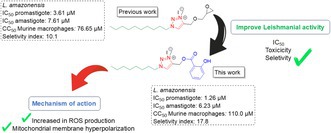

## 1 Introduction

Neglected tropical diseases (NTDs) are considered a world health issue caused by a diversity of pathogens, including the *Leishmania* spp. parasite (World Health Organization 2024). At least 20 species infect humans, which are transmitted through the bite of an infected female phlebotomine sandfly. In the middle of several neglected diseases, leishmaniasis is ranked among the top 10 NTDs worldwide due to its high prevalence, affecting over 12 million people (Pan American Health Organization, n.d.). Leishmaniasis manifests with various symptoms such as anemia, fever, weight loss, and skin and mucous membranes lesions resembling ulcers. Depending on the symptoms present, it is classified into three forms: cutaneous, mucocutaneous, and visceral leishmaniasis (World Health Organization 2023).

Treatment for leishmaniasis typically involves commercially available drugs. Some of the medications on the market include miltefosine, pentamidine, amphotericin B, and meglumine antimoniate. However, these drugs are associated with issues such as lack of selectivity, toxicity, adverse effects in numerous organs, resistance, and extended treatment (Gupta et al. 2021). Due to this, the development of new compounds that can minimize these inconveniences is necessary.

**SCHEME 1:**
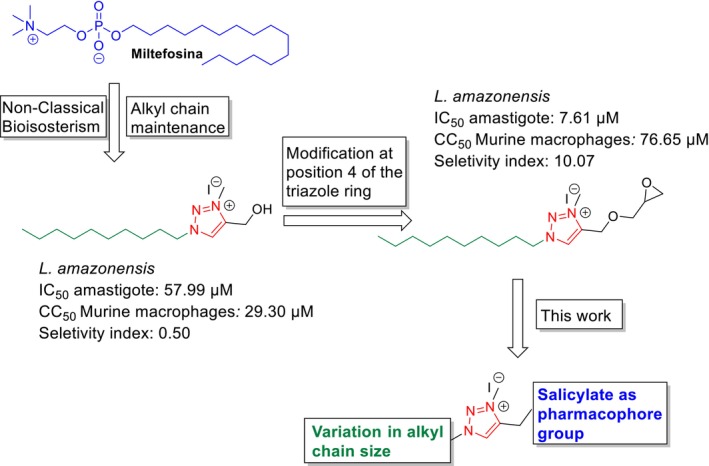
Rational development of compounds in this work.

Recent studies conducted by our research group have led to the development of synthetic compounds derived from 1,2,3‐triazoles, demonstrating promising antileishmanial (Das Chagas Almeida et al. 2022; Glanzmann et al. 2021; Meinel et al. 2020; Stroppa et al. 2017) and antitumor (De Paula et al. 2024; Ramos et al. 2022) activities. The design of these compounds was guided by a non‐classical bioisosterism approach, using miltefosine, the only oral drug approved for the treatment of leishmaniasis, as a structural reference. Specifically, the presence of aliphatic chains at the 1‐position of the triazole ring was inspired by the alkylphosphocholine tail of miltefosine, aiming to mimic its amphiphilic properties and membrane‐targeting capabilities. These investigations underscore the versatility of the 1,2,3‐triazolic nucleus, attributable to its capacity for functionalization and/or coupling of pharmacophoric groups to the alkyl chain, as well as the potential for structural variation through the formation of organic salts. Despite the notable biological activity exhibited by these compounds, their cytotoxicity remains a significant concern. In a study conducted by Meinel et al. (2020), the replacement of the hydroxyl group at position 4 of the 1,2,3‐triazole ring by an epoxide group resulted in reduced cytotoxicity in murine macrophage cells and a significant increase in activity against the amastigote forms of *L. amazonensis*, as shown in Scheme 1. This structural modification, therefore, represents a viable strategy to mitigate the high cytotoxicity and improve the biological activity in this class of compounds.

One of the reaction pathways that enables the derivatization of alkyl 1,2,3‐triazole into other compounds involves the formation of esters from carboxylic acid. Specifically, salicylic esters, or salicylates as they are also known, are reported to possess a range of diverse biological activities described in the literature, such as cholinesterase inhibition (Wang et al. 2023), antitumor (Liu et al. 2020; Tsepaeva et al. 2021), antifungal (Wodnicka et al. 2017), anti‐inflammatory (Koca et al. 2023; Vasconcelos et al. 2012), and antibacterial agents (Zhi‐Hua Guo et al. 2015). However, their derivatives remain underexplored in terms of their antileishmanial activity.

Salicylates are derivatives of salicylic acid (SA), a naturally occurring phenolic compound synthesized by plants, known for its diverse medicinal applications and significant pharmaceutical potential through its derivatives. SA exhibits antiseptic and anti‐inflammatory properties and is commonly used to treat conditions such as acne, psoriasis, and warts. Additionally, it serves as a key intermediate in the synthesis of various medications. Notably, aspirin, an acetylated derivative of SA, possesses analgesic and anti‐inflammatory properties and ranks among the most widely produced medications globally, with annual production reaching several thousand tons (Ekinci et al. 2011; Sambyal and Singh 2021). Due to its pharmacological importance, developing compounds containing the SA moiety has become highly interesting.

*Leishmania amazonensis*, chosen as the *Leishmania* species for this study, is considered an important public health problem in Latin America, mainly in the Amazon region. This species is associated with various forms of cutaneous leishmaniasis, and *L*. *amazonensis* isolates from patients with different clinical manifestations exhibited genetic heterogeneity (Barral et al. 1991; de Oliveira et al. 2007). For example, *L. amazonensis* is the main etiological agent of diffuse cutaneous leishmaniasis (DLC) in some regions of South America, causing multiple non‐ulcerative skin lesions. It is also associated with mucocutaneous forms and even visceral leishmaniasis in humans, dogs, and animal models (da Silva et al. 2018; Tolezano et al. 2007; Valdivia et al. 2017). Furthermore, *L. amazonensis* occurs in broad areas overlapping with *L. infantum*, classically related to the visceral form, considered fatal if untreated (da Silva et al. 2018; Tolezano et al. 2007; Valdivia et al. 2017).

This work is grounded in this approach, facilitating the formation of novel salicylic esters at position 4 of the 1,2,3‐triazole ring, followed by the formation of their organic salts. Subsequently, the study evaluates their antileishmanial activity, specifically against the *L. amazonensis* species, with a particular focus on a potential mode of action. Although 1,2,3‐triazole derivatives have been previously explored for antileishmanial purposes, the combination of substitution patterns presented in this study is, to our knowledge, without precedent.

## 2 Materials and Methods

### 2.1 Chemistry

Sigma‐Aldrich provided all starting materials and reagents, which were used without further purification. A Bruker Avance III 500 MHz NMR spectrometer was employed to record ^1^H and ^13^C spectra, with TMS (δ = 0 ppm) as the reference for chemical shifts. Melting point data were determined using an MQAPF‐301‐Microquímica digital device. FTIR spectra were acquired on a Bruker Alpha using an ATR module. The high‐resolution mass spectrometer used was the Q‐Exactive Plus (Thermo Scientific) with electrospray ionization.

#### 2.1.1 General Procedures for the Preparation of Alkyne (**1**)

In a round‐bottom flask, salicylic acid (1 equivalent) was dissolved in acetone. Potassium carbonate (1 equivalent) was then added, and the mixture was stirred for 1 h at room temperature. Afterward, propargyl bromide (1 equivalent) was added to the reaction mixture, which was stirred overnight at room temperature. The solvent was removed using a rotary evaporator. The crude product was purified by column chromatography using CH_2_Cl_2_ as the eluent. After evaporation of the solvent, the alkyne derived from salicylic acid was obtained (An et al. 2020). The product was obtained as a white crystal (yield = 89%). m.p. = 51.9°C–53.8°C. ^1^H NMR (500 MHz, CDCl_3_) δ 10.51 (*s*, 1H), 7.87 (*d*, *J* = 8.0 Hz, 1H), 7.46 (*t*, *J* = 7.8 Hz, 1H), 6.98 (*d*, *J* = 8.4 Hz, 1H), 6.88 (*t*, *J* = 7.6 Hz, 1H), 4.94 (*s*, 2H), 2.57 (*s*, 1H). ^13^C NMR (125 MHz, CDCl_3_) δ 169.4, 161.8, 136.2, 130.2, 119.4, 117.7, 111.9, 75.7, 52.8.

#### 2.1.2 General Procedures for the Preparation of Alkyl Azides

The synthesis of alkyl azides was carried out as described by Stroppa et al. (2017).

#### 2.1.3 General Procedure for the Preparation of Triazoles (**2–6**)

In a round‐bottom flask, 1 equivalent of intermediate (**1**) and 1 equivalent of the respective alkyl azide were dissolved in CH_2_Cl_2_. An aqueous solution containing 0.1 equivalent of CuSO_4_.5H_2_O and 0.6 equivalent of sodium ascorbate was then added to the mixture. The reaction was stirred at room temperature for 72 h. Purification began by removing the solvent via rotary evaporation, followed by washing the resulting solid with ethyl acetate. The organic phase was evaporated to obtain the crude reaction product, which was further purified by column chromatography using a hexane/ethyl acetate mixture in an 8:2 ratio as the eluent.

##### 2.1.3.1 (1‐Octyl‐1H‐1,2,3‐Triazol‐4‐Yl)methyl 2‐Hydroxybenzoate (**2**)

The product was obtained as an orange solid (yield = 88%). m.p. = 53.0°C–55.3°C. HRMS‐ESI: *m/z* calculated for C_18_H_26_N_3_O_3_ [M + H]^+^ 332.1974, found: 332.1963. IR (ATR), ν (cm^−1^): 2926 (aromatic C—H), 2854 (aliphatic C—H), 1613 (C=N), 1483 (N=N), 1673 (C=O ester), 1296 (C—O ester). ^1^H NMR (500 MHz, CDCl_3_) δ 10.63 (*s*, 1H), 7.79 (dd, *J* = 8.0, 1.8 Hz, 1H), 7.68 (*s*, 1H), 7.40 (ddd, *J* = 8.7, 7.2, 1.8 Hz, 1H), 6.92 (*d*, *J* = 8.4 Hz, 1H), 6.81 (*t*, *J* = 7.6 Hz, 1H), 5.46 (*s*, 2H), 4.32 (*t*, *J* = 7.3 Hz, 2H), 1.88 (*q*, *J* = 7.3 Hz, 2H), 1.22 (bs, 10H), 0.86 (*t*, *J* = 6.8 Hz, 3H). ^13^C NMR (125 MHz, CDCl_3_) δ 169.9, 161.6, 142.1, 135.8, 130.0, 119.2, 117.5, 112.1, 58.3, 50.4, 31.6, 30.1, 28.9, 28.8, 26.4, 22.5, 13.9.

##### 2.1.3.2 (1‐Decyl‐1H‐1,2,3‐Triazol‐4‐Yl)methyl 2‐Hydroxybenzoate (**3**)

The product was obtained as an orange solid (yield = 92%). m.p. = 60.7°C–62.8°C. HRMS‐ESI: *m/z* calculated for C_20_H_30_N_3_O_3_ [M + H]^+^ 360.2287, found: 360.2275. IR (ATR), ν (cm^−1^): 2917 (aromatic C—H), 2850 (aliphatic C—H), 1610 (C=N), 1480 (N=N), 1673 (C=O ester), 1248 (C—O ester). ^1^H NMR (500 MHz, CDCl_3_) δ 10.66 (*s*, 1H), 7.84 (*d*, *J* = 8.3 Hz, 1H), 7.67 (*s*, 1H), 7.45 (ddd, *J* = 8.5, 7.2, 1.6 Hz, 1H), 6.97 (*d*, *J* = 8.4 Hz, 1H), 6.85 (*t*, *J* = 7.6 Hz, 1H), 5.50 (*s*, 2H), 4.35 (*t*, *J* = 7.2 Hz, 2H), 1.91 (*q*, *J* = 6.6 Hz, 2H), 1.24 (bs, 14H), 0.87 (*t*, *J* = 6.9 Hz, 3H). ^13^C NMR (125 MHz, CDCl_3_) δ 170.1, 161.9, 142.4, 136.1, 130.3, 124.0, 119.4, 117.7, 58.6, 50.7, 32.0, 30.4, 29.6, 29.5, 29.4, 29.1, 26.6, 22.8, 14.2.

##### 2.1.3.3 (1‐Dodecyl‐1H‐1,2,3‐Triazol‐4‐Yl)methyl 2‐Hydroxybenzoate (**4**)

The product was obtained as an orange solid (yield = 92%). m.p. = 64.6°C–67.3°C. HRMS‐ESI: *m/z* calculated for C_22_H_34_N_3_O_3_ [M + H]^+^ 388.2600, found: 388.2593. IR (ATR), ν (cm^−1^): 2924 (aromatic C—H), 2854 (aliphatic C—H), 1612 (C=N), 1460 (N=N), 1676 (C=O ester), 1248 (C—O ester). ^1^H NMR (500 MHz, CDCl_3_) δ 10.66 (*s*, 1H), 7.84 (*d*, *J* = 8.1 Hz, 1H), 7.67 (*s*, 1H), 7.45 (*t*, *J* = 7.9 Hz, 1H), 6.97 (*d*, *J* = 7.8 Hz, 1H), 6.85 (*t*, *J* = 7.6 Hz, 1H), 5.50 (*s*, 2H), 4.35 (*t*, *J* = 7.3 Hz, 2H), 1.92 (*q*, *J* = 6.9 Hz, 2H), 1.24 (bs, 18H), 0.87 (*t*, *J* = 6.7 Hz, 3H). ^13^C NMR (125 MHz, CDCl_3_) δ 170.4, 162.1, 142.6, 136.4, 130.6, 124.3, 119.7, 118.0, 112.6, 58.8, 50.9, 32.3, 30.7, 30.0, 29.9, 29.8, 29.7, 29.4, 26.9, 23.1, 14.5.

##### 2.1.3.4 (1‐Tetradecyl‐1H‐1,2,3‐Triazol‐4‐Yl)methyl 2‐Hydroxybenzoate (**5**)

The product was obtained as an orange solid (yield = 91%). m.p. = 75.1°C–77.9°C. HRMS‐ESI: *m/z* calculated for C_24_H_38_N_3_O_3_ [M + H]^+^ 416.2913, found: 416.2898. IR (ATR), ν (cm^−1^): 22,916 (aromatic C—H), 2846 (aliphatic C—H), 1612 (C=N), 1480 (N=N), 1675 (C=O ester), 1246 (C—O ester). ^1^H NMR (500 MHz, CDCl_3_) δ 10.66 (*s*, 1H), 7.84 (*d*, *J* = 8.0 Hz, 1H), 7.67 (*s*, 1H), 7.45 (ddd, *J* = 8.6, 7.4, 1.8 Hz, 1H), 6.97 (*d*, *J* = 8.4 Hz, 1H), 6.86 (*t*, *J* = 7.6 Hz, 1H), 5.50 (*s*, 2H), 4.35 (*t*, *J* = 7.4 Hz, 2H), 1.91 (*q*, *J* = 7.42 Hz, 2H), 1.24 (bs, 22H), 0.87 (*t*, *J* = 6.9 Hz, 3H). ^13^C NMR (125 MHz, CDCl_3_) δ 170.1, 161.9, 142.4, 136.1, 130.3, 124.0, 119.4, 117.7, 112.3, 58.5, 50.7, 32.1, 30.4, 29.8, 29.8, 29.7, 29.6, 29.5, 29.5, 29.1, 26.6, 22.8, 14.2.

##### 2.1.3.5 (1‐Hexadecyl‐1H‐1,2,3‐Triazol‐4‐Yl)methyl 2‐Hydroxybenzoate (**6**)

The product was obtained as an orange solid (yield = 94%). m.p. = 78.3°C–79.6°C. HRMS‐ESI: *m/z* calculated for C_26_H_42_N_3_O_3_ [M + H]^+^ 444.3226, found: 444.3217. IR (ATR), ν (cm^−1^): 2917 (aromatic C—H), 2850 (aliphatic C—H), 1612 (C=N), 1463 (N=N), 1675 (C=O ester), 1248 (C—O ester).

^1^H NMR (500 MHz, CDCl_3_) δ 10.66 (*s*, 1H), 7.84 (*d*, *J* = 8.2 Hz, 1H), 7.67 (*s*, 1H), 7.45 (*t*, *J* = 7.9 Hz, 1H), 6.97 (*d*, *J* = 8.4 Hz, 1H), 6.85 (*t*, *J* = 7.6 Hz, 1H), 5.50 (*s*, 2H), 4.35 (*t*, *J* = 7.3 Hz, 2H), 1.91 (*q*, *J* = 7.2 Hz, 2H), 1.25 (bs, 26H), 0.87 (*t*, *J* = 6.8 Hz, 3H). ^13^C NMR (125 MHz, CDCl_3_) δ 169.9, 161.6, 142.1, 135.9, 130.1, 123.7, 119.1, 117.5, 112.1, 58.3, 50.4, 30.2, 29.6, 29.6, 29.5, 29.9, 29.2, 28.9, 26.4, 22.6, 14.0.

#### 2.1.4 General Procedure for the Preparation of Triazolium Salts (**7–11**)

In a round‐bottom flask, 1 equivalent of the corresponding 1,2,3‐triazole derivatives and 4 equivalents of methyl iodide were dissolved in acetonitrile. The reaction mixture was then refluxed under stirring for 48 h. After the completion of the reaction, the solvent was evaporated under reduced pressure. The resulting crude product was purified by column chromatography using a CH_2_Cl_2_/MeOH gradient as the eluent, affording the corresponding triazolium salts.

##### 2.1.4.1 1‐Octyl‐4‐(((2‐Hydroxybenzoyl)oxy)methyl)‐3‐Methyl‐1H‐1,2,3‐Triazol‐3‐Ium Iodide (**7**)

The product was obtained as a brown oil (yield = 44%). HRMS‐ESI: *m/z* calculated for C_19_H_28_N_3_O_3_^+^ [M + H]^+^ 346.2130, found: 346.2121. IR (ATR), ν (cm^−1^): 2926 (aromatic C—H), 2856 (aliphatic C—H), 1613 (C=N), 1483 (N=N), 1679 (C=O ester), 1293 (C—O ester). ^1^H NMR (500 MHz, CDCl_3_) δ 10.10 (*s*, 1H), 9.39 (*s*, 1H), 7.83 (dd, *J* = 7.9, 1.8 Hz, 1H), 7.44 (ddd, *J* = 8.7, 7.2, 1.8 Hz, 1H), 6.91 (*d*, *J* = 8.4 Hz, 1H), 6.87 (*t*, *J* = 7.6 Hz, 1H), 5.85 (*s*, 2H), 4.67 (*t*, *J* = 7.6 Hz, 2H), 4.51 (*s*, 3H), 2.01 (*q*, *J* = 7.6 Hz, 2H), 1.20 (bs, 10H), 0.81 (*t*, *J* = 6.8 Hz, 3H). ^13^C NMR (125 MHz, CDCl_3_) δ 169.1, 161.8, 138.7, 136.9, 132.0, 130.3, 119.8, 117.8, 110.9, 55.2, 54.7, 40.2, 31.6, 29.4, 28.9, 28.8, 26.2, 22.5, 14.0.

##### 2.1.4.2 1‐Decyl‐4‐(((2‐Hydroxybenzoyl)oxy)methyl)‐3‐Methyl‐1H‐1,2,3‐Triazol‐3‐Ium Iodide (**8**)

The product was obtained as a brown oil (yield = 35%). HRMS‐ESI: *m/z* calculated for C_21_H_32_N_3_O_3_^+^ [M + H]^+^ 374.2443, found: 374.2431. IR (ATR), ν (cm^−1^): 2921 (aromatic C—H), 2850 (aliphatic C—H), 1613 (C=N), 1483 (N=N), 1683 (C=O ester), 1293 (C—O ester). ^1^H NMR (500 MHz, CDCl_3_) δ 10.11 (*s*, 1H), 9.41 (*s*, 1H), 7.84 (*d*, *J* = 8.0 Hz, 1H), 7.46 (*t*, *J* = 7.8 Hz, 1H), 6.93 (*d*, *J* = 8.4 Hz, 1H), 6.88 (*t*, *J* = 7.6 Hz, 1H), 5.87 (*s*, 2H), 4.69 (*t*, *J* = 7.6 Hz, 2H), 4.59 (*s*, 3H), 2.02 (*q*, *J* = 7.7 Hz, 2H), 1.21 (bs, 18H), 0.84 (*t*, *J* = 6.8 Hz, 3H). ^13^C NMR (125 MHz, CDCl_3_) δ 169.4, 162.0, 138.99, 137.1, 132.2, 130.3, 119.9, 118.0, 111.0, 55.2, 54.8, 40.0, 31.9, 29.5, 29.5, 29.4, 29.3, 29.0, 26.3, 22.8, 14.2.

##### 2.1.4.3 1‐Dodedecyl‐4‐(((2‐Hydroxybenzoyl)oxy)methyl)‐3‐Methyl‐1H‐1,2,3‐Triazol‐3‐Ium Iodide (**9**)

The product was obtained as a brown oil (yield = 54%). HRMS‐ESI: *m/z* calculated for C_23_H_36_N_3_O_3_^+^ [M + H]^+^ 402.2757, found: 402.2746. IR (ATR), ν (cm^−1^): 2920 (aromatic C—H), 2851 (aliphatic C—H), 1613 (C=N), 1463 (N=N), 1686 (C=O ester), 1295 (C—O ester). ^1^H NMR (500 MHz, CDCl_3_) δ10.13 (*s*, 1H), 9.35 (*s*, 1H), 7.85 (dd, *J* = 8.1, 1.8 Hz, 1H), 7.48 (ddd, *J* = 8.7, 7.2, 1.8 Hz, 1H), 6.96 (*d*, *J* = 8.4 Hz, 1H), 6.90 (*t*, *J* = 7.7 Hz, 1H), 5.87 (*s*, 2H), 4.68 (*t*, *J* = 7.6 Hz,21H), 4.52 (*s*, 3H), 2.03 (*q*, *J* = 7.5 Hz, 2H), 1.22 (bs, 18H), 0.86 (*t*, *J* = 6.9 Hz, 3H). ^13^C NMR (125 MHz, CDCl_3_) δ 169.1, 161.7, 138.7, 136.8, 131.9, 130.1, 119.7, 117.8, 110.7, 54.9, 54.6, 39.8, 31.7, 29.4, 29.3, 29.2, 29.2, 29.1, 28.7, 26.0, 22.5, 13.9.

##### 2.1.4.4 1‐Tetradecyl‐4‐(((2‐Hydroxybenzoyl)oxy)methyl)‐3‐Methyl‐1H‐1,2,3‐Triazol‐3‐Ium Iodide (**10**)

The product was obtained as a brown oil (yield = 52%). HRMS‐ESI: *m/z* calculated for C_25_H_40_N_3_O_3_^+^ [M + H]^+^ 430.3070, found: 430.3060. IR (ATR), ν (cm^−1^): 2917 (aromatic C—H), 2850 (aliphatic C—H), 1613 (C=N), 1465 (N=N), 1685 (C=O ester), 1296 (C—O ester). ^1^H NMR (500 MHz, CDCl_3_) δ 10.13 (*s*, 1H), 9.36 (*s*, 1H), 7.86 (*d*, *J* = 8.1 Hz, 1H), 7.49 (*t*, *J* = 7.9 Hz, 1H), 6.97 (*d*, *J* = 8.4 Hz, 1H), 6.91 (*t*, *J* = 7.6 Hz, 1H), 5.88 (*s*, 2H), 4.68 (*t*, *J* = 7.5 Hz, 2H), 4.53 (*s*, 3H), 2.05 (*q*, *J* = 7.5 Hz, 2H), 1.23 (bs, 22H), 0.86 (*t*, *J* = 6.8 Hz, 3H). ^13^C NMR (125 MHz, CDCl_3_) δ 169.1, 161.8, 138.8, 136.8, 131.9, 130.1, 119.7, 117.8, 110.7, 55.0, 54.6, 39.8, 31.8, 29.5, 29.5, 29.5, 29.4, 29.3, 29.2, 29.2, 28.7, 26.1, 22.5, 14.0.

##### 2.1.4.5 1‐Hexadecyl‐4‐(((2‐Hydroxybenzoyl)oxy)methyl)‐3‐Methyl‐1H‐1,2,3‐Triazol‐3‐Ium Iodide (**11**)

The product was obtained as a brown oil (yield = 45%). HRMS‐ESI: *m/z* calculated for C_27_H_44_N_3_O_3_^+^ [M + H]^+^ 458.3383, found: 458.3369. IR (ATR), ν (cm^−1^): 2917 (aromatic C—H), 2849 (aliphatic C—H), 1615 (C=N), 1468 (N=N), 1683 (C=O ester), 1296 (C—O ester). ^1^H NMR (500 MHz, CDCl_3_) δ 10.13 (*s*, 1H), 9.40 (*s*, 1H), 7.86 (*d*, *J* = 8.0 Hz, 1H), 7.49 (*t*, *J* = 7.9 Hz, 1H), 6.96 (*d*, *J* = 8.4 Hz, 1H), 6.91 (*t*, *J* = 7.6 Hz, 1H), 5.89 (*s*, 2H), 4.69 (*t*, *J* = 7.6 Hz, 2H), 4.54 (*s*, 3H), 2.04 (*q*, *J* = 7.6 Hz, 2H), 1.24 (bs, 26H), 0.86 (*t*, *J* = 6.8 Hz, 3H). ^13^C NMR (125 MHz, CDCl_3_) δ 169.3, 162.0, 139.0, 137.1, 132.1, 130.3, 119.9, 118.0, 111.0, 55.3, 54.8, 40.1, 32.0, 29.8, 29.8, 29.7, 29.6, 29.5, 29.5, 29.4, 26.3, 22.8, 14.2.

### 2.2 Biological Experiments

#### 2.2.1 Parasites

Promastigotes of *L. amazonensis* (IFLA/BR/67/PH8) were cultivated in brain heart infusion (BHI‐ Kasvi, São José dos Pinhais, PR, Brazil) supplemented with bovine hemin (Sigma‐Aldrich, St. Louis, MO, USA), folic acid (Sigma‐Aldrich, St. Louis, MO, USA), inactivated fetal bovine serum (Cultilab, São Paulo, Brazil), and penicillin/streptomycin solution (Sigma‐Aldrich, St. Louis, MO, USA). The cells were kept at 25°C in a BOD incubator.

#### 2.2.2 Activity Against Promastigotes of *L. amazonensis*

Promastigotes of *L. amazonensis* in the logarithmic phase, containing 2 × 10^6^ cells/mL, were cultured in 96‐well plates using different doses of the compounds (3.125–100.000 μM), and the cell viability was assessed using the colorimetric method 72 h after treatment began, employing 3‐(4,5‐dimethylthiazol‐2‐yl)‐2‐5‐diphenyltetrazolium (MTT) (Sigma‐Aldrich, St. Louis, MO, USA; Mosmann 1983). The viability of parasites was measured *using a spectrophotometer* (Multiskan EX‐Thermo Electron Corporation, Vantaa, Finland) at 570 nm. The values were expressed as an inhibitory concentration capable of inhibiting 50% (IC_50_) of cell growth by using GraFit 5 software (Erithacus Software Ltda). Miltefosine (Cayman Chemical Company, Ann Arbor, MI, USA) and amphotericin B (Cristalia, São Paulo, SP, Brazil) were used as reference drugs. All assays were run in triplicate across three separate trials.

#### 2.2.3 Activity Against Intracellular Amastigote Forms of *L. amazonensis*

Peritoneal macrophages were obtained from BALB/c mice previously inoculated with 3% thioglycollate medium (da Trindade Granato et al. 2018). The macrophages (2 × 10^6^ cells/mL) were then allowed to adhere in 24‐well culture plates for 16 h at 37°C in a 5% CO_2_ atmosphere. After rinsing with phosphate‐buffered saline (PBS), the adhered macrophages were infected with stationary‐phase promastigotes of *L. amazonensis* at a 10:1 parasite‐to‐macrophage ratio for 4 h at 33°C. After several washes to remove non‐adherent promastigotes, the cells were either left untreated (negative control) or treated with the compounds (3.125–100.000 μM). Following a 72 h treatment period, the infected macrophages were fixed in ethanol, stained with Giemsa, and 200 macrophages were counted to determine the number of intracellular amastigotes. The GraFit software was used to determine the IC_50_, which was the outcome of three separate trials conducted in duplicate.

#### 2.2.4 Cytotoxicity Activity Against Mammalian Cells

After being prepared as previously mentioned, murine peritoneal macrophages obtained from BALB/c mice were either left untreated (negative control) or treated with the compounds (3.125–100.000 μM). Using a spectrophotometer (Multiskan EX, Thermo Electron Corporation, Vantaa, Finland) set to 570 nm, cell viability was assessed after 72 h of treatment using the MTT method (Mosmann 1983). A cytotoxic concentration for 50% of the cell population (CC_50_) was used to express the results. The reference medication was miltefosine (Cayman Chemical Company, Ann Arbor, MI, USA). The assays were performed in three independent experiments in duplicate. The selectivity index was calculated by IC_50_ amastigotes/CC_50_.

#### 2.2.5 Cytotoxicity in Human Red Blood Cells

Blood cells were centrifuged at 1500 rpm, the plasma and leukocytes were discarded, and the erythrocytes were washed in PBS. Then, the cells were diluted to a concentration of 1%, plated in 96‐well plates, and untreated or treated with the compound **7** at different concentrations (0.09–200.00 μM). The plate was incubated for 24 h at 37°C, and then centrifuged at 2000 rpm for 1 min. The supernatant was collected, and the absorbance (Abs) was determined in a spectrophotometer (Multiskan EX, Thermo Electron Corporation, Vantaa, Finland) at 540 nm. Untreated cells were considered the negative control. For the positive control, 0.2% saponin in deionized water was used. Amphotericin B (Cristália, São Paulo, SP, Brazil) was used as the reference drug (Granato et al. 2024).

### 2.3 Mitochondrial Functional Assays

#### 2.3.1 Reactive Oxygen Species Production in Promastigotes and Intracellular Amastigotes of *L. amazonensis*

To measure ROS and mitochondrial ROS (superoxide) levels, *L. amazonensis* promastigotes (5 × 10^6^ cells) were exposed to compound **7** (1.25 and 2.50 μM) for 6 and 24 h, following the methodology described by da Trindade Granato et al. (2018). Before the incubation, cells were washed with PBS and stained with 1 mM H_2_DCFDA (Invitrogen, Waltham, MA, USA) to measure total ROS. Fluorescence was measured after a 30‐min incubation with the dye using a fluorimeter (FLx800, BioTek Instruments Inc., Winooski, VT, USA) at an excitation/emission wavelength of 485/528 nm. As a positive control, cells were treated with 62.5 μM H_2_O_2_. To quantify mitochondrial superoxide, the MitoSOX Red indicator (Invitrogen, Molecular Probes, Eugene, Oregon, USA) was employed. *Leishmania amazonensis* promastigotes were treated with compound **7** (1.25 and 2.50 μM) for 6 and 24 h at 25°C. Cells were labeled with MitoSOX Red indicator at 5 μM and incubated for 20 min. Promastigotes incubated with antimycin A (10 μM) were used as the positive control. Fluorescence intensity was evaluated in a spectrofluorometer (FLx800, BioTek Instruments Inc., Winooski, VT, USA) at 540/600 nm of excitation/emission, respectively.

#### 2.3.2 Determination of Mitochondrial Membrane Potential

To evaluate mitochondrial membrane potential (MMP), *L. amazonensis* promastigotes (5 × 10^6^ cells) were exposed to compound **7** (1.25 μM and 2.50 μM) for 6 and 24 h. After incubation, the cells were washed with PBS and stained with 5.00 μM JC‐1 (Coimbra et al. 2019). After a 40‐min incubation with JC‐1, the cells were washed with Hank's solution, and fluorescence was measured using a fluorimeter (FLx800, BioTek Instruments Inc., Winooski, VT, USA) at excitation/emission wavelengths of 485/528 nm and 540/600 nm to detect fluorescence from monomers and J‐aggregates, respectively. As a positive control, cells were treated with 10.00 μM FCCP (Sigma‐Aldrich, St. Louis, MO, USA). MMP was assessed by calculating the ratio of fluorescence emitted by J‐aggregates to that of monomers.

#### 2.3.3 Evaluation of Cell Membrane Integrity

To evaluate cell membrane integrity, *L. amazonensis* promastigotes (5 × 10^6^ cells) were treated with compound **7** (1.25 and 2.50 μM) for 24 h. After incubation, the cells were washed with PBS and stained with 0.20 μg/mL of propidium iodide (PI) (Sigma‐Aldrich, St. Louis, MO, USA) (da Trindade Granato et al. 2018). Following a 15‐min incubation with PI, fluorescence was measured using a fluorimeter (FLx800) at an excitation/emission wavelength of 540/600 nm. Cells exposed to heat at 65°C for 10 min were used as a positive control.

### 2.4 Prediction of Physicochemical Properties and ADME Properties

The pkCSM web tool (Pires et al. 2015) (https://biosig.lab.uq.edu.au/pkcsm) was utilized to predict the physicochemical, ADME, and toxicity properties of compound **7**. Predictions were performed using the default pkCSM parameters, which apply graph‐based signatures to estimate multiple pharmacokinetic endpoints. The evaluated parameters include water solubility, gastrointestinal absorption, skin permeability, CYP450 interactions, AMES toxicity, and renal OCT2 substrate. These outputs were used to support the assessment of the compound's predicted pharmacokinetic and safety profile.

### 2.5 Ethical Aspects

#### 2.5.1 Animals

Female BALB/c mice (4–6 weeks, 15–20 g) were obtained from the Reproductive Biology Center of the Federal University of Juiz de Fora (CBR/UFJF). Animals were randomly housed in groups of five per cage under standard housing conditions for a research experiment (24°C; 50% ± 5% humidity) with a 12‐h light and 12‐h dark cycle. Free access to food and water was allowed. A veterinarian performed daily welfare monitoring. All procedures were approved by the Committee on the Use of Animals (CEUA/UFJF) under protocols #007/2018 and #008/2018.

#### 2.5.2 Humans

Research involving human participants was approved by the Ethics in Research with Human Beings Committee (CEP/UFJF), under the approval #3.697.188.

## 3 Results and Discussion

### 3.1 Chemistry

**SCHEME 2:**
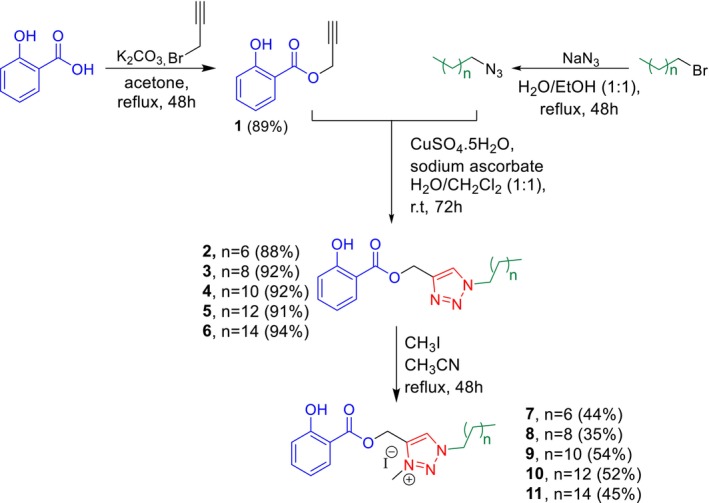
Synthesis of 1,2,3‐triazole 4‐substituted salicylic ester derivatives and their salts.

According to Scheme 2, a novel series of 1,2,3‐triazole compounds 4‐substituted from salicylic ester and their salts were synthesized via a convergent approach.

Initially, the alkyne **1** was prepared by esterification of salicylic acid using a strategy involving in situ deprotonation of the acid and formation of its potassium salt, and subsequent nucleophilic substitution reaction with propargyl bromide. Concurrently, alkyl azides were synthesized through nucleophilic substitution reactions between sodium azide and the respective alkyl bromides. The compounds **2–6** were then obtained in high yields via a copper‐catalyzed cycloaddition reaction between the corresponding alkyl azides and alkyne **1**. Finally, the 1,2,3‐triazolium salts (**7–11**) were synthesized in moderate yields through *N*‐alkylation reaction of the corresponding compounds **2–6** with methyl iodide.

An analysis of the ^1^H NMR data confirms the successful synthesis of the triazolium salts and their corresponding intermediates.

For the alkyne precursor **1**, the ^1^H NMR spectrum displays the expected resonances of the propargylic fragment. The terminal methine proton appears as a triplet, whereas the propargylic methylene proton resonates as a doublet, both exhibiting a small coupling constant (*J* = 2 Hz), consistent with the characteristic coupling pattern of terminal alkynes. In the 6–8 ppm region, characteristic of aromatic protons, the expected signals of the salicylate moiety are observed.

The formation of 1,2,3‐triazole derivatives **2–6** is supported by the presence of a singlet at approximately 7.7 ppm, attributed to the hydrogen atom present in the triazole ring, confirming the construction of the 1,4‐disubstituted triazole core. Additional resonances between 0 and 4.6 ppm correspond to the aliphatic chain protons, while the aromatic protons of the salicylate group appear within the 6–8 ppm region.

The triazolium salts **7–11** exhibit ^1^H NMR spectra that closely resemble those of their respective precursors. However, salt formation is unambiguously evidenced by the emergence of a new singlet integrating for three protons at approximately 4.6 ppm, attributed to the methyl group introduced by the *N*‐alkylation reaction on the triazole ring. A second defining feature supporting triazolium formation is the pronounced downfield shift of the triazole ring proton, which appears near 9.4 ppm due to the positive charge localized on the heteroaromatic system.

The experimental data on compounds characterization are reported in the Section 2 and Supporting Information S1.

### 3.2 Biological

In vitro antileishmanial activity against both promastigote and amastigote forms of *L. amazonensis* after 72 h of treatment with the compounds was evaluated as detailed in the Section 2. The IC_50_ and CC_50_ values of the newly synthesized compounds, as well as those of miltefosine and amphotericin B (reference drugs used for leishmaniasis treatment), are presented in Table 1. The results indicate that neutral compounds (**2–6**) were inactive against both biological forms of *L. amazonensis* (IC_50_ > 100.00 μM). Regarding activity against amastigotes, compounds **7**, **8**, and **10** exhibited the lowest IC_50_ values (6.23 ± 0.35 μM, 2.49 ± 0.50 μM, and 5.92 ± 2.24 μM, respectively). Interestingly, we note that: (i) the ionic compounds (**7–11**), derived from the neutral compounds, were able to inhibit the growth of promastigotes and intracellular amastigotes at low concentrations, with IC_50_ values ranging from 1.08 ± 0.18 μM to 12.36 ± 1.86 μM; (ii) additionally, compounds **7–11** were more active against promastigote form and demonstrated similar activity against intracellular amastigotes when compared to miltefosine (IC_50_ values of 15.05 ± 2.08 μM and 11.50 ± 1.79 μM, respectively). The variation in drug sensitivity between the biological forms of the parasite is well‐documented in the literature, including for drugs used in leishmaniasis treatment, such as pentavalent antimonials, miltefosine, and amphotericin B (Vermeersch et al. 2009). This variation may be attributed not only to the fact that amastigotes are intracellular forms but also to intrinsic characteristics of the parasite (Ait Maatallah et al. 2022).

**TABLE 1:** In vitro antileishmanial and cytotoxicity activities of synthesized 1,2,3‐triazole 4‐substituted salicylic ester.

Compounds	Promastigote IC_50_ (μM)	Amastigote IC_50_ (μM)	Cytotoxicity CC_50_ (μM)	SI
2	> 100.00	> 100.00	> 150.00	—
3	> 100.00	> 100.00	70.16 ± 14.15	
4	> 100.00	> 100.00	> 150.00	—
5	> 100.00	> 100.00	> 150.00	—
6	> 100.00	> 100.00	> 150.00	—
7	1.26 ± 0.27	6.23 ± 0.35	111.01 ± 34.52	17.81
8	1.08 ± 0.18	2.49 ± 0.50	0.31 ± 0.22	0.12
9	1.58 ± 0.12	6.31 ± 1.86	1.34 ± 0.32	0.21
10	1.55 ± 0.34	5.92 ± 2.24	2.37 ± 0.82	0.40
11	1.72 ± 0.02	12.36 ± 1.86	4.01 ± 1.12	0.32
Miltefosine	15.05 ± 2.08	11.50 ± 1.79	151.81 ± 0.03	13.20
Am B	0.10 ± 0.02	85.81 ± 30.40	0.71 ± 0.22	858.10

*Note:* IC_50_: Concentration that inhibits by 50% the growth of *L. amazonensis* promastigotes and amastigotes. CC_50_: Concentration capable of causing cytotoxic effect on 50% of peritoneal murine macrophages. IS (Selectivity Index): CC_50_ of peritoneal murine macrophages/IC_50_ *L. amazonensis* amastigotes. Am B: amphotericin B. These results correspond to the average of three experiments performed in duplicate, calculated by the program GraFit 5 software. Miltefosine was used as a positive control.

**FIGURE 1:**
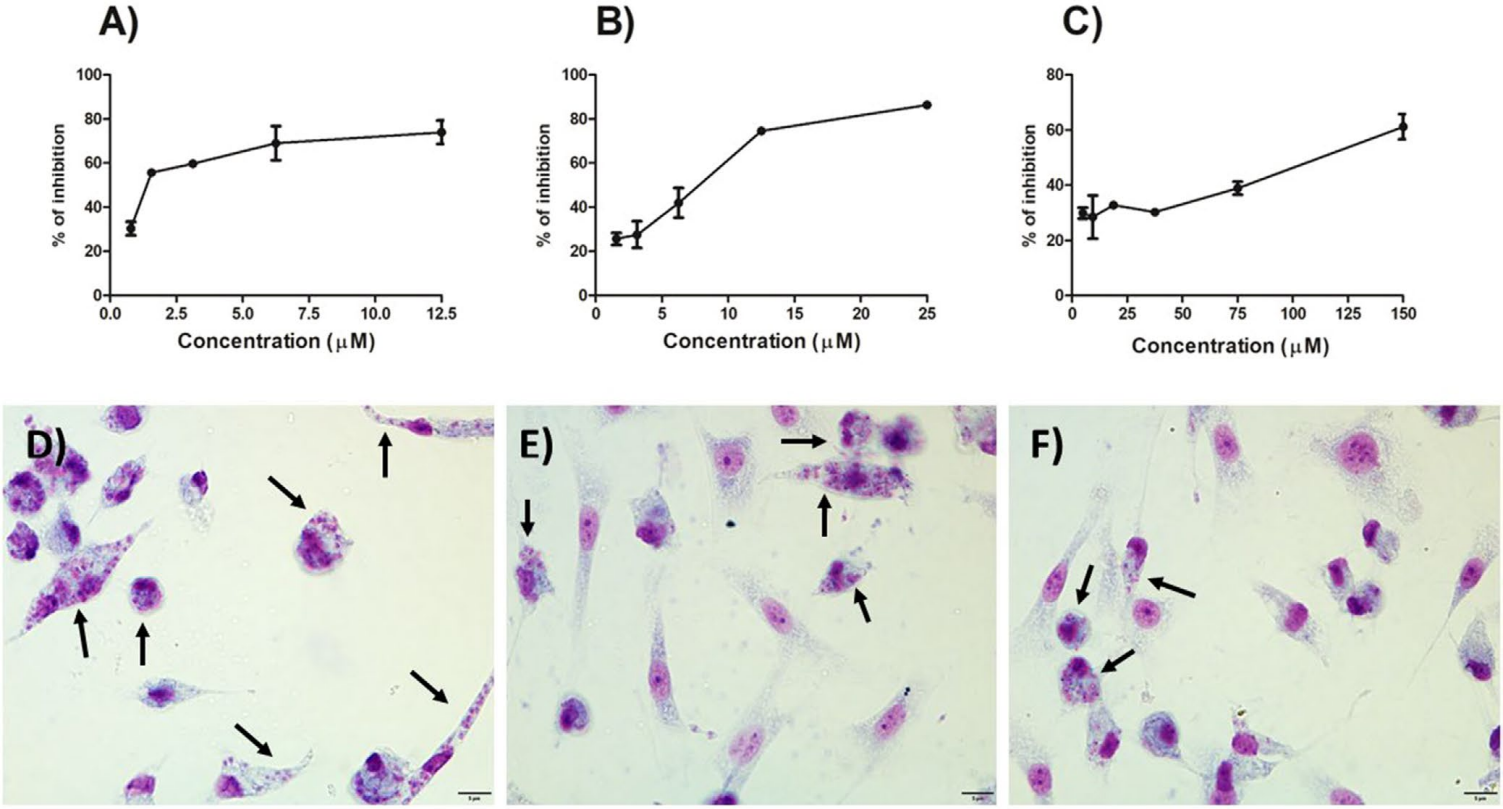
In vitro effect of compound **7** against *L. amazonensis* and on macrophages after 72 h. (A) Promastigotes of *L. amazonensis* were treated with several concentrations of compound **7** (0.78–12.50 μM). (B) Peritoneal macrophages were infected with *L. amazonensis* and treated with compound **7** (1.56–25.00 μM). (C) Macrophages were treated with compound **7** (4.68–150.00 μM). (D) Representative images of *Giemsa‐staining of L. amazonensis*‐infected macrophages (negative control). (E) Macrophages infected with *L. amazonensis* and treated with compound **7** at 6.23 μM. (F) Macrophages infected with *L. amazonensis* and treated with compound **7** at 12.48 μM. The results are from one experiment, representative of a total of three.

To evaluate the toxicity in mammalian cells, peritoneal murine macrophages were chosen as the model (Table 1). Macrophages play a dual role in *Leishmania* spp. infection, acting both as human defense cells and as hosts for the parasite. While the neutral compounds (**2–6**) were inactive against the parasite and did not exhibit notable toxicity to macrophages (CC_50_ > 70.00 μM), the ionic compounds (**7–11**) exhibited toxicity to mammalian cells, which compromised their selectivity. For instance, compound **8** displayed the highest activity against both promastigote and amastigote forms of *L. amazonensis* (IC_50_ values of 1.08 ± 0.18 μM and 2.49 ± 0.50 μM, respectively), but it was also the most cytotoxic (CC_50_ = 0.31 ± 0.22 μM), with a selectivity index (SI) of 0.12. In contrast, compound **7** was less cytotoxic (CC_50_ = 111.01 ± 34.52 μM) and, with an IC_50_ value of 6.23 ± 0.35 μM against amastigote forms, emerged as the most promising compound in the new series, with an SI of 17.81, slightly higher than that of miltefosine (SI = 13.20). Figure 1 shows a potent inhibitory effect of compound **7** against promastigotes and intracellular amastigotes, even at the lowest concentrations tested (1A and 1C, respectively). Regarding the toxicity on macrophages, compound **7** exhibited a dose‐dependent effect, being more deleterious at high concentrations (Figure 1B). In the same figure, it can be seen that macrophages infected with *L. amazonensis* and treated with compound **7** (Figure 1E,F) showed a reduced parasite load and did not display visible signs of suffering induced by the treatment, maintaining the morphological characteristics of negative control (Figure 1D).

The urgent need for new antileishmanial agents is not only based on potency, but also on the growing concern about parasite resistance, which significantly compromises their long‐term efficacy (Rastrojo et al. 2018). In this context, the development of new compounds, even with comparable activity profiles, as observed by the IC_50_ and SI range values of compound **7** and miltefosine, is strategically important to diversify the therapeutic arsenal and reduce the risk of resistance (Gupta et al. 2021). In this sense, our triazole‐based analogue, although similar in vitro potency, may present itself as a viable alternative, especially in regions where treatment efficacy is compromised.

The compounds synthesized in this study differ primarily in the length of their alkyl chains and in the comparison between neutral compounds and their corresponding salts. When correlating structural variations with antileishmanial activity, compound **7**, a methylated organic salt with an eight‐carbon chain, emerges as the active derivative with the shortest carbon chain. The difference in activity among the evaluated compounds is mainly attributable to their cytotoxicity. Compound **7** exhibited the lowest toxicity; however, for compounds **8–11**, an increase in carbon chain length was associated with a reduction in toxicity, despite a lack of selectivity in some cases.

Considering the findings of Meinel et al. (2020), the 4‐epoxy‐substituted derivative 1‐decyl‐3‐methyl‐4‐((oxiran‐2‐ylmethoxy)methyl)‐1*H*‐1,2,3‐triazol‐3‐ium iodide was identified as the most active compound in their study, demonstrating approximately 2.5‐fold lower cytotoxicity and 7.5‐fold higher activity against amastigote forms of the parasite compared to its 4‐hydroxy‐substituted precursor. In the present work, the substitution of the epoxy group with a salicylate moiety, along with a reduction of the alkyl chain to eight carbons, results in compound **7** exhibiting 3.8‐fold lower cytotoxicity and 9.3‐fold greater activity against the intracellular form compared to the 4‐hydroxy‐substituted precursor previously described (Meinel et al. 2020). These findings highlight the impact of structural modifications on improving both selectivity and efficacy in the class of compounds.

**FIGURE 2:**
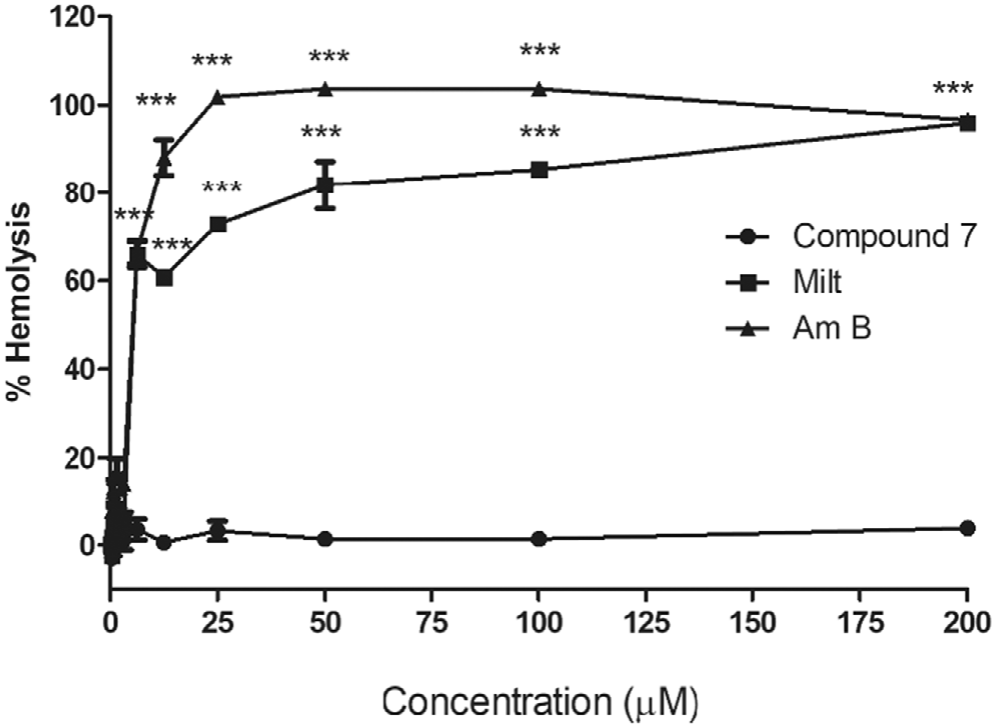
Hemolytic assay. Human erythrocytes were washed and incubated with varying concentrations of compound **7**, miltefosine (Milt), and amphotericin B (Am B). Untreated cells were considered as a negative control. The graph was built in the GraFit 8.0 software. Results expressed as mean ± standard deviation of three independent experiments. One‐way ANOVA followed by Bonferroni's post hoc test. a means statistical difference compared to the untreated control group. *p* < 0.001 (***), significant difference compared with the negative control.

Then, to obtain more information about its toxicity, the hemolytic capacity of compound **7** was evaluated in human erythrocytes. Thus, after 24 h of treatment, compound **7** showed low hemolytic capacity (3.82%) at the maximum concentration tested (200.00 μM). In contrast, amphotericin B and miltefosine, currently used as antileishmanial drugs, showed a strong hemolytic effect at all concentrations, with an average of 95.9% hemolysis at the same concentration cited above (Figure 2). These findings highlight compound **7** as a potentially selective antileishmanial agent. In addition, oral treatment employing miltefosine or amphotericin B by intravenous (IV) route may cause hemolytic anemia as a side effect. Therefore, the absence of hemolytic activity found for compound **7** can be considered promising (Scariot et al. 2017).

### 3.3 Studies to Investigate the Effects of Compound **7** on Mitochondrial Function

Based on the selective and antileishmanial activity of the 1,2,3‐triazole compounds 4‐substituted with salicylic esters and their salts, compound **7** was selected for further investigation of its impact on parasite physiology, particularly its potential to alter mitochondrial function. For all assays, and based on the IC_50_ value, two concentrations of this compound were selected: 1.25 μM and 2.50 μM, corresponding to 1 and 2 times the IC_50_ values against *L. amazonensis* promastigotes, respectively.

*Leishmania* spp. is an intracellular parasite that resides within macrophages. These host cells induce an oxidative burst to attempt to eliminate the parasite, as well as to contribute to the immune response. Oxidative stress, including increased production of ROS, can also result from stress conditions such as heat shock, drug treatment, and nutrient deprivation, all of which ultimately lead to cell death. Several drugs used to treat leishmaniasis, such as amphotericin B, pentavalent antimonials, miltefosine, and amodiaquine, exert their antileishmanial activity through mechanisms associated with oxidative stress (Alpizar‐Sosa et al. 2022; Antinarelli et al. 2023; Ponte‐Sucre et al. 2017).

**FIGURE 3:**
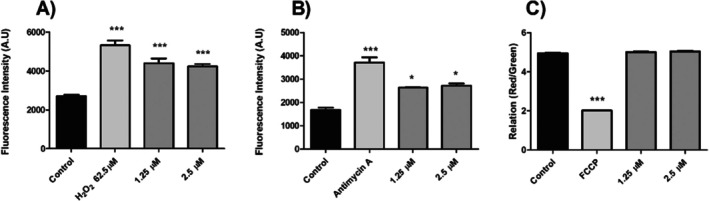
Evaluation of ROS levels, membrane integrity, and mitochondrial membrane potential in promastigotes of *L. amazonensis* treated with compound **7** at IC_50_ and 2× IC_50_, at 6 h. (A) The evaluation of total ROS levels was performed by using the fluorescent dye H_2_DCFDA. H_2_O_2_ was used as a positive control. (B) For the evaluation of mitochondrial superoxide, parasites are stained with MitoSox. Antimycin A was used as a positive control. (C) For the evaluation of mitochondrial membrane potential (MMP), parasites staining with JC‐1 were used to measure the ∆Ψm. FCC was used as a positive control. In all assays, fluorescence was measured fluorometrically, and in (A, B), the data were expressed as the fluorescence intensity in arbitrary units (a.u.) of the means of three independent experiments. *** *p* < 0.001 and * *p* < 0.05, significant difference compared with the negative control.

**FIGURE 4:**
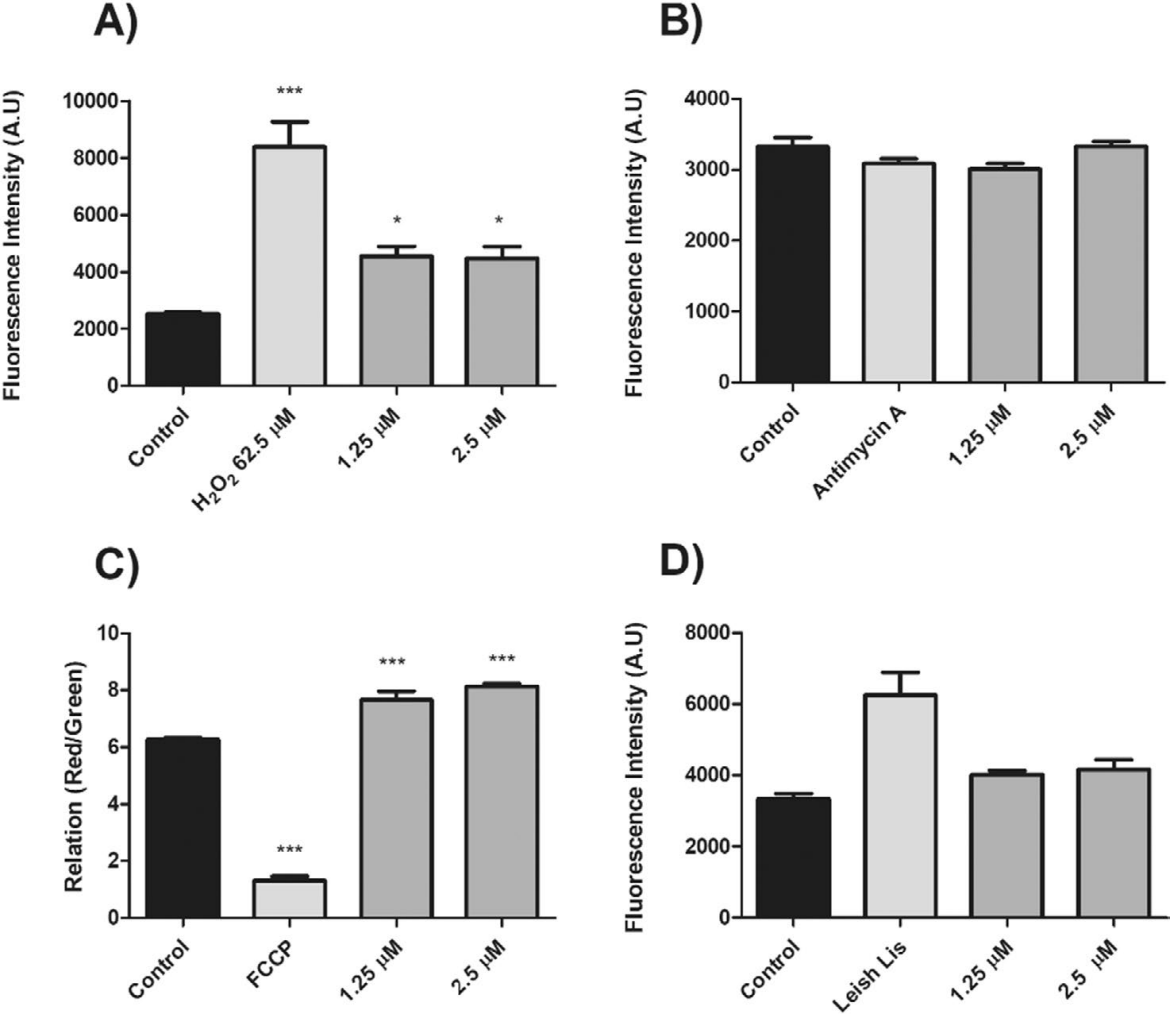
Evaluation of ROS levels, mitochondrial membrane potential, and membrane integrity in promastigotes of *L. amazonensis* treated with compound **7** at IC_50_ and 2× IC_50_, at 24 h. (A) The evaluation of total ROS levels was performed by using the fluorescent dye H_2_DCFDA. H_2_O_2_ was used as a positive control. (B) For the evaluation of mitochondrial superoxide, parasites are stained with MitoSox. Antimycin A was used as a positive control. (C) For the evaluation of mitochondrial membrane potential (MMP), parasites staining with JC‐1 were used to measure the ∆Ψm. FCC was used as a positive control. (D) Evaluation of membrane integrity by using PI. Leish Lis: promastigotes of *L. amazonensis* lysed by heat at 65°C were used as a positive control. In all assays, fluorescence was measured fluorometrically, and in (A–C), the data were expressed as the fluorescence intensity in arbitrary units (a.u.) of means of three independent experiments.*** *p* < 0.001 and * *p* < 0.05 significant difference compared with the negative control.

With this in mind, we sought to determine whether compound **7** could alter the levels of total ROS within the parasite's cells as well as the superoxide radical, a specific type of ROS produced within the mitochondrion. We also performed mitochondrial membrane potential assays to assess the functionality of this organelle. To investigate the sequence of these events, all assays were performed at early (6 h) and late (24 h) time points following treatment. At 6 h (Figure 3), the results showed a significant increase in mitochondrial superoxide (57.68% and 62.54% in IC_50_ value and 2× IC_50_ value, respectively) and total ROS (62.81% and 56.54% in IC_50_ value and 2× IC_50_ value, respectively) levels, without alterations in mitochondrial membrane potential (Figure 3A–C). At 24 h (Figure 4), an increase was observed in total ROS (79.87% and 77.03% at the IC_50_ value and 2× IC_50_ value, respectively) and a mitochondrial membrane hyperpolarization (22.00% and 29.66% at the IC_50_ value and 2× IC_50_ value, respectively), as demonstrated by our JC‐1 assays (Figure 4A,C, respectively). However, no alteration in mitochondrial superoxide levels was observed in *L. amazonensis* promastigotes after 24 h of treatment (Figure 4B).

These findings support the hypothesis that compound **7** exerts its antileishmanial effect through mitochondrial dysfunction, with mitochondrial superoxide generation being an early event. The absence of mitochondrial superoxide at 24 h suggests that this radical was consumed in subsequent events, leading to mitochondrial hyperpolarization and the production of other ROS, including hydrogen peroxide. Similar patterns have been reported for other compounds that target mitochondria of *Leishmania* spp., where enhanced ROS production leads to bioenergetic disruption (de Souza et al. 2025). Taken together, these findings support the hypothesis that mitochondrial dysfunction plays a central role in the antileishmanial mechanism of compound **7**. This oxidative burst can damage crucial biomolecules within the parasite, such as proteins, lipids, and DNA, impairing its ability to evade the host immune system and induce the death of *Leishmania* spp. Furthermore, in trypanosomatids, including *Leishmania* spp., the parasite's mitochondrion is unique and, therefore, considered an excellent target for the discovery of new drugs against leishmaniasis. This organelle is responsible for the majority of the parasite's energy demands, and the mitochondrial respiratory chain is the main cellular source of ROS (Kathuria et al. 2014).

Since both ROS production and hyperpolarization are associated with necrosis, we further investigated changes in membrane integrity using propidium iodide (PI) staining, which serves as an indicator of necrotic cell death. Our data revealed no significant alterations (*p* > 0.05) after PI staining (Figure 4D), excluding necrosis and suggesting the involvement of other cell death mechanisms (Albuquerque et al. 2020). So far, our results reinforce that the protozoan mitochondrion is affected and that the imbalance in this organelle, which is unique to *Leishmania* spp., causes damage to the survival and multiplication of the parasite.

### 3.4 In Silico*‐*Predicted ADMET Profile

Despite the importance of a good biological activity, a compound's overall efficiency also depends on having an acceptable ADMET profile, as evaluating its effects and potential risks in the human body is crucial. Given that in vitro and in vivo pharmacokinetics tests are labor‐intensive and costly, in silico predictions have emerged as a practical and valuable alternative for early‐stage compound evaluation (Cheng et al. 2012; Davis and Riley 2004).

In this study, the pkCSM web tool was employed to computationally estimate the physicochemical, pharmacokinetics, drug‐likeness, and toxicity properties of compound **7**, with the predicted values summarized in Tables 2 and 3.

**TABLE 2:** Physicochemical properties of compound **7**.

Predicted properties	Compound **7**	Reference
MW (g/mol)	473.35	< 500
HBA	5	≤ 10
HBD	1	≤ 5
RB	10	≤ 10
Log P	0.14	≤ 5

Abbreviations: HBA, number of hydrogen bond acceptor; HBD, number of hydrogen bond donor; Log P, partition coefficient between n‐octanol and water; MW, molecular weight; RB, rotatable bonds.

**TABLE 3:** ADMET predict property of compound **7**.

Absorption	GI absorption (%)	70.73
	Skin permeability (log Kp)	−2.85
Metabolism	CYP2D6 substrate	No
	CYP3A4 substrate	Yes
	CYP1A2 inhibitor	No
	CYP2C19 inhibitor	Yes
	CYP2C9 inhibitor	No
	CYP2D6 inhibitor	No
	CYP3A4 inhibitor	No
Toxicity	AMES toxicity	No
Excretion	Renal OCT2 substrate	No

Abbreviations: BBB, blood–brain barrier; GI, gastrointestinal; OCT2, organic cation transporter.

Based on predicted physicochemical parameters (Table 2), compound **7** complies with Lipinski's rules of five criteria, including acceptable values for molecular weight, hydrogen bond acceptors and donors, rotatable bonds, and lipophilicity (log *P*), suggesting a favorable oral bioavailability (Lipinski 2004).

The predicted pharmacokinetics profile (Table 3) suggests high gastrointestinal absorption, with an estimated absorption rate above 70%, which is generally associated with good oral bioavailability (Wadapurkar et al. 2018). It also shows low predicted skin permeability (log Kp < −2.5), which may be beneficial for reducing dermal absorption (Saritha et al. 2024). These computational estimates suggest the potential for effective intracellular accumulation, which could contribute to the compound's pharmacological activity (Elmeliegy et al. 2020; Nguyen et al. 2021).

Predictions regarding metabolic behavior indicate that compound **7** may act as an inhibitor of the CYP2C19 and CYP3A4 isoforms of the cytochrome P450 enzyme family. This is particularly significant, as CYP3A4 is involved in the metabolism of approximately 50% of clinically used drugs, potentially affecting drug–drug interactions (Hakkola et al. 2020). However, this prediction remains to be confirmed through experimental assays.

Toxicity‐related predictions also suggest a favorable safety profile. For instance, the compound tested negative in the in silico AMES test, indicating a low likelihood of mutagenicity (Xu et al. 2012). Additionally, the compound is predicted not to interact with organic cation transporters (OCTs), which are involved in renal excretion. This may suggest a primary hepatic route of elimination, which could be advantageous in patients with impaired kidney function (Motohashi and Inui 2013). Nonetheless, these computational findings are intended to guide compound prioritization and must be interpreted with caution until validated by experimental data.

Taken together, the biological results and in silico predictions support the potential of compound **7** as a promising candidate and encourage future studies against other *Leishmania* species.

## 4 Conclusion

In this study, a novel series of 4‐substituted 1,2,3‐triazole compounds with salicylic esters and their salts was synthesized and evaluated for activity against *L. amazonensis*. Among the compounds tested, the shortest‐chain methylated organic salt, compound **7**, stood out as the most selective, surpassing miltefosine in terms of selectivity index and efficacy against the amastigote form of the parasite. Furthermore, studies on the biological effects of the compound indicate that its activity is associated with alterations in mitochondrial function, evidenced by increased production of total and mitochondrial ROS, as well as hyperpolarization of the mitochondrial membrane potential. In silico analyses predicted a favorable pharmacokinetic profile for compound **7**, including high gastrointestinal absorption, low permeability to the central nervous system, and absence of mutagenic or cardiotoxic effects, indicating a promising safety profile.

## Funding

This work was supported the Fundação de Amparo à Pesquisa do Estado de Minas Gerais (FAPEMIG) (APQ‐01646‐23; RED‐00198‐23). Ari Sérgio de Oliveira Lemos was a grant recipient of Coordenação de Aperfeiçoamento de Pessoal de Nível Superior (CAPES) (88887.835464/2023‐00). Elaine Soares Coimbra is a grant recipient of Conselho Nacional de Desenvolvimento Científico e Tecnológico (CNPq).

## Conflicts of Interest

The authors declare no conflicts of interest.

## Supporting information


**Data S1:** cbdd70227‐sup‐0001‐Figures.docx.

## Data Availability

The data that supports the findings of this study are available in the Supporting Information S1 of this article.

## References

[cbdd70227-bib-0001] Ait Maatallah, I. , K. Akarid , and M. Lemrani . 2022. “Tissue Tropism: Is It an Intrinsic Characteristic of Leishmania Species?” Acta Tropica 232: 106512. 10.1016/j.actatropica.2022.106512. 35568069

[cbdd70227-bib-0002] Albuquerque, R. D. D. G. , A. P. Oliveira , C. Ferreira , et al. 2020. “Anti‐Leishmania Amazonensis Activity of the Terpenoid Fraction From Eugenia Pruniformis Leaves.” Anais da Academia Brasileira de Ciências 92, no. 4: e20201181. 10.1590/0001-3765202020201181. 33295583

[cbdd70227-bib-0003] Alpizar‐Sosa, E. A. , N. R. B. Ithnin , W. Wei , et al. 2022. “Amphotericin B Resistance in Leishmania Mexicana: Alterations to Sterol Metabolism and Oxidative Stress Response.” PLoS Neglected Tropical Diseases 16, no. 9: e0010779. 10.1371/journal.pntd.0010779. 36170238 PMC9581426

[cbdd70227-bib-0004] An, J. , R. Pedrazzani , M. Monari , M. Marin‐Luna , C. S. Lopez , and M. Bandini . 2020. “Site‐Selective Synthesis of 1,3‐Dioxin‐3‐Ones via a Gold(i) Catalyzed Cascade Reaction.” Chemical Communications 56, no. 56: 7734–7737. 10.1039/D0CC02703K. 32582892

[cbdd70227-bib-0005] Antinarelli, L. M. R. , V. Midlej , E. D. S. da Silva , E. A. F. Coelho , A. D. da Silva , and E. S. Coimbra . 2023. “Exploring the Repositioning of the Amodiaquine as Potential Drug Against Visceral Leishmaniasis: The In Vitro Effect Against Leishmania Infantum Is Associated With Multiple Mechanisms, Involving Mitochondria Dysfunction, Oxidative Stress and Loss of Cell Cycle Control.” Chemico‐Biological Interactions 371: 110333. 10.1016/j.cbi.2022.110333. 36592711

[cbdd70227-bib-0006] Barral, A. , D. Pedral‐Sampaio , G. Grimaldi , et al. 1991. “Leishmaniasis in Bahia, Brazil: Evidence That Leishmania Amazonensis Produces a Wide Spectrum of Clinical Disease.” American Journal of Tropical Medicine and Hygiene 44, no. 5: 536–546. 10.4269/ajtmh.1991.44.536. 2063957

[cbdd70227-bib-0007] Cheng, F. , W. Li , Y. Zhou , et al. 2012. “admetSAR: A Comprehensive Source and Free Tool for Assessment of Chemical ADMET Properties.” Journal of Chemical Information and Modeling 52, no. 11: 3099–3105. 10.1021/ci300367a. 23092397

[cbdd70227-bib-0008] Coimbra, E. S. , M. V. Nora de Souza , M. S. Terror , A. C. Pinheiro , and J. da Trindade Granato . 2019. “Synthesis, Biological Activity, and Mechanism of Action of New 2‐Pyrimidinyl Hydrazone and N‐Acylhydrazone Derivatives, a Potent and New Classes of Antileishmanial Agents.” European Journal of Medicinal Chemistry 184: 111742. 10.1016/j.ejmech.2019.111742. 31605866

[cbdd70227-bib-0009] da Silva, C. , F. de Souza , K. da Silva , et al. 2018. “Leishmania Amazonensis Isolated From Human Visceral Leishmaniasis: Histopathological Analysis and Parasitological Burden in Different Inbred Mice.” Histology and Histopathology 33, no. 7: 705–716. 29345298 10.14670/HH-11-965

[cbdd70227-bib-0010] da Trindade Granato, J. , J. A. dos Santos , S. L. Calixto , et al. 2018. “Novel Steroid Derivatives: Synthesis, Antileishmanial Activity, Mechanism of Action, and In Silico Physicochemical and Pharmacokinetics Studies.” Biomedicine & Pharmacotherapy 106: 1082–1090. 10.1016/j.biopha.2018.07.056. 30119174

[cbdd70227-bib-0011] Das Chagas Almeida, A. , R. S. Meinel , Y. L. Leal , et al. 2022. “Functionalized 1,2,3‐Triazolium Salts as Potential Agents Against Visceral Leishmaniasis.” Parasitology Research 121, no. 5: 1389–1406. 10.1007/s00436-022-07431-9. 35169883

[cbdd70227-bib-0012] Davis, A. M. , and R. J. Riley . 2004. “Predictive ADMET Studies, the Challenges and the Opportunities.” Current Opinion in Chemical Biology 8, no. 4: 378–386. 10.1016/j.cbpa.2004.06.005. 15288247

[cbdd70227-bib-0013] de Oliveira, J. P. C. , F. Fernandes , A. K. Cruz , et al. 2007. “Genetic Diversity of Leishmania Amazonensisstrains Isolated in Northeastern Brazil as Revealed by DNA Sequencing, PCR‐Based Analyses and Molecular Karyotyping.” Kinetoplastid Biology and Disease 6, no. 1: 5. 10.1186/1475-9292-6-5. 17584940 PMC1919383

[cbdd70227-bib-0014] De Paula, W. T. , N. Glanzmann , I. K. Da Costa Nunes , et al. 2024. “Synthesis and Evaluation of New 1,2,3‐Triazole Alkyl Derivatives and Their Salts Against Breast Cancer Cells.” ChemistrySelect 9, no. 6: e202303321. 10.1002/slct.202303321.

[cbdd70227-bib-0015] de Souza, A. R. , L. M. R. Antinarelli , A. S. d. O. Lemos , et al. 2025. “Multiple Mechanisms of Action of a Triazole‐Derived Salt Against Leishmania Amazonensis: Apoptosis‐Like Death and Autophagy.” Chemico‐Biological Interactions 409: 111409. 10.1016/j.cbi.2025.111409. 39922522

[cbdd70227-bib-0016] Ekinci, D. , M. Şentürk , and Ö. İ. Küfrevioğlu . 2011. “Salicylic Acid Derivatives: Synthesis, Features and Usage as Therapeutic Tools.” Expert Opinion on Therapeutic Patents 21, no. 12: 1831–1841. 10.1517/13543776.2011.636354. 22098318

[cbdd70227-bib-0017] Elmeliegy, M. , M. Vourvahis , C. Guo , and D. D. Wang . 2020. “Effect of P‐Glycoprotein (P‐Gp) Inducers on Exposure of P‐Gp Substrates: Review of Clinical Drug‐Drug Interaction Studies.” Clinical Pharmacokinetics 59, no. 6: 699–714. 10.1007/s40262-020-00867-1. 32052379 PMC7292822

[cbdd70227-bib-0018] Glanzmann, N. , L. M. R. Antinarelli , I. K. da Costa Nunes , et al. 2021. “Synthesis and Biological Activity of Novel 4‐Aminoquinoline/1,2,3‐Triazole Hybrids Against Leishmania Amazonensis.” Biomedicine & Pharmacotherapy 141: 111857. 10.1016/j.biopha.2021.111857. 34323702

[cbdd70227-bib-0019] Granato, J. d. T. , E. T. da Silva , A. S. d. O. Lemos , et al. 2024. “4‐Quinolinylhydrazone Analogues Kill *Leishmania* (*Leishmania*) *Amazonensis* by Inducing Apoptosis and Mitochondria‐Dependent Pathway Cell Death.” Chemical Biology & Drug Design 103, no. 5: e14535. 10.1111/cbdd.14535. 38772877

[cbdd70227-bib-0020] Guo, Z.‐H. , Y. Yin , C. Wang , et al. 2015. “Design, Synthesis and Molecular Docking of Salicylic Acid Derivatives Containing Metronidazole as a New Class of Antimicrobial Agents.” Bioorganic & Medicinal Chemistry 23, no. 18: 6148–6156. 10.1016/j.bmc.2015.07.075. 26304108

[cbdd70227-bib-0021] Gupta, O. , T. Pradhan , R. Bhatia , and V. Monga . 2021. “Recent Advancements in Anti‐Leishmanial Research: Synthetic Strategies and Structural Activity Relationships.” European Journal of Medicinal Chemistry 223: 113606. 10.1016/j.ejmech.2021.113606. 34171661

[cbdd70227-bib-0022] Hakkola, J. , J. Hukkanen , M. Turpeinen , and O. Pelkonen . 2020. “Inhibition and Induction of CYP Enzymes in Humans: An Update.” Archives of Toxicology 94, no. 11: 3671–3722. 10.1007/s00204-020-02936-7. 33111191 PMC7603454

[cbdd70227-bib-0023] Kathuria, M. , A. Bhattacharjee , K. V. Sashidhara , S. P. Singh , and K. Mitra . 2014. “Induction of Mitochondrial Dysfunction and Oxidative Stress in Leishmania Donovani by Orally Active Clerodane Diterpene.” Antimicrobial Agents and Chemotherapy 58, no. 10: 5916–5928. 10.1128/AAC.02459-14. 25070112 PMC4187897

[cbdd70227-bib-0024] Koca, M. , B. Anıl , B. Nişancı , Y. Bayır , Z. Ercan , and E. Özakar . 2023. “Synthesis of New Ester Derivatives of Salicylic Acid and Evaluation of Their COX Inhibitory Potential.” Chemistry & Biodiversity 20, no. 1: e202200509. 10.1002/cbdv.202200509. 36514919

[cbdd70227-bib-0025] Lipinski, C. A. 2004. “Lead‐ and Drug‐Like Compounds: The Rule‐Of‐Five Revolution.” Drug Discovery Today: Technologies 1, no. 4: 337–341. 10.1016/j.ddtec.2004.11.007. 24981612

[cbdd70227-bib-0026] Liu, R. , X. Deng , Y. Peng , et al. 2020. “Synthesis and Biological Evaluation of Novel 5,6,7‐Trimethoxy Flavonoid Salicylate Derivatives as Potential Anti‐Tumor Agents.” Bioorganic Chemistry 96: 103652. 10.1016/j.bioorg.2020.103652. 32059154

[cbdd70227-bib-0027] Meinel, R. S. , A. d. C. Almeida , P. H. F. Stroppa , N. Glanzmann , E. S. Coimbra , and A. D. da Silva . 2020. “Novel Functionalized 1,2,3‐Triazole Derivatives Exhibit Antileishmanial Activity, Increase in Total and Mitochondrial‐ROS and Depolarization of Mitochondrial Membrane Potential of Leishmania Amazonensis.” Chemico‐Biological Interactions 315: 108850. 10.1016/j.cbi.2019.108850. 31634447

[cbdd70227-bib-0028] Mosmann, T. 1983. “Rapid Colorimetric Assay for Cellular Growth and Survival: Application to Proliferation and Cytotoxicity Assays.” Journal of Immunological Methods 65, no. 1–2: 55–63. 10.1016/0022-1759(83)90303-4. 6606682

[cbdd70227-bib-0029] Motohashi, H. , and K. Inui . 2013. “Organic Cation Transporter OCTs (SLC22) and MATEs (SLC47) in the Human Kidney.” AAPS Journal 15, no. 2: 581–588. 10.1208/s12248-013-9465-7. 23435786 PMC3675737

[cbdd70227-bib-0030] Nguyen, T.‐T.‐L. , V.‐A. Duong , and H.‐J. Maeng . 2021. “Pharmaceutical Formulations With P‐Glycoprotein Inhibitory Effect as Promising Approaches for Enhancing Oral Drug Absorption and Bioavailability.” Pharmaceutics 13, no. 7: 1103. 10.3390/pharmaceutics13071103. 34371794 PMC8309061

[cbdd70227-bib-0031] Pan American Health Organization . n.d. “Leishmaniasis.”

[cbdd70227-bib-0032] Pires, D. E. V. , T. L. Blundell , and D. B. Ascher . 2015. “pkCSM: Predicting Small‐Molecule Pharmacokinetic and Toxicity Properties Using Graph‐Based Signatures.” Journal of Medicinal Chemistry 58, no. 9: 4066–4072. 10.1021/acs.jmedchem.5b00104. 25860834 PMC4434528

[cbdd70227-bib-0033] Ponte‐Sucre, A. , F. Gamarro , J.‐C. Dujardin , et al. 2017. “Drug Resistance and Treatment Failure in Leishmaniasis: A 21st Century Challenge.” PLoS Neglected Tropical Diseases 11, no. 12: e0006052. 10.1371/journal.pntd.0006052. 29240765 PMC5730103

[cbdd70227-bib-0034] Ramos, J. P. , M. A. L. Abdel‐Salam , D. A. B. Nobre , et al. 2022. “Acute Toxicity and Antitumor Potential of 1,3,4‐Trisubstituted‐1,2,3‐Triazole dhmtAc‐Loaded Liposomes on a Triple‐Negative Breast Cancer Model.” Archiv Der Pharmazie 355, no. 9: 2200004. 10.1002/ardp.202200004. 35621705

[cbdd70227-bib-0035] Rastrojo, A. , R. García‐Hernández , P. Vargas , et al. 2018. “Genomic and Transcriptomic Alterations in Leishmania Donovani Lines Experimentally Resistant to Antileishmanial Drugs.” International Journal for Parasitology: Drugs and Drug Resistance 8, no. 2: 246–264. 10.1016/j.ijpddr.2018.04.002. 29689531 PMC6039315

[cbdd70227-bib-0036] Sambyal, K. , and R. V. Singh . 2021. “Production of Salicylic Acid; a Potent Pharmaceutically Active Agent and Its Future Prospects.” Critical Reviews in Biotechnology 41, no. 3: 394–405. 10.1080/07388551.2020.1869687. 33618601

[cbdd70227-bib-0037] Saritha, K. , M. Alivelu , and M. Mohammad . 2024. “Drug‐Likeness Analysis, In Silico ADMET Profiling of Compounds in Kedrostis Foetidissima (Jacq.) Cogn, and Antibacterial Activity of the Plant Extract.” In Silico Pharmacology 12, no. 2: 67. 10.1007/s40203-024-00240-1. 39050777 PMC11264488

[cbdd70227-bib-0038] Scariot, D. B. , E. A. Britta , A. L. Moreira , et al. 2017. “Induction of Early Autophagic Process on Leishmania Amazonensis by Synergistic Effect of Miltefosine and Innovative Semi‐Synthetic Thiosemicarbazone.” Frontiers in Microbiology 8: 255. 10.3389/fmicb.2017.00255. PMC5318461 28270805

[cbdd70227-bib-0039] Stroppa, P. H. F. , L. M. R. Antinarelli , A. M. L. Carmo , J. Gameiro , E. S. Coimbra , and A. D. da Silva . 2017. “Effect of 1,2,3‐Triazole Salts, Non‐Classical Bioisosteres of Miltefosine, on Leishmania Amazonensis.” Bioorganic & Medicinal Chemistry 25, no. 12: 3034–3045. 28433512 10.1016/j.bmc.2017.03.051

[cbdd70227-bib-0040] Tolezano, J. E. , S. R. B. Uliana , H. H. Taniguchi , et al. 2007. “The First Records of Leishmania (Leishmania) Amazonensis in Dogs (*Canis familiaris*) Diagnosed Clinically as Having Canine Visceral Leishmaniasis From Araçatuba County, São Paulo State, Brazil.” Veterinary Parasitology 149, no. 3–4: 280–284. 10.1016/j.vetpar.2007.07.008. 17720321

[cbdd70227-bib-0041] Tsepaeva, O. V. , T. I. Salikhova , L. R. Grigor'eva , et al. 2021. “Synthesis and In Vitro Evaluation of Triphenylphosphonium Derivatives of Acetylsalicylic and Salicylic Acids: Structure‐Dependent Interactions With Cancer Cells, Bacteria, and Mitochondria.” Medicinal Chemistry Research 30, no. 4: 925–939. 10.1007/s00044-020-02674-6.

[cbdd70227-bib-0042] Valdivia, H. O. , L. V. Almeida , B. M. Roatt , et al. 2017. “Comparative Genomics of Canine‐Isolated Leishmania (Leishmania) Amazonensis From an Endemic Focus of Visceral Leishmaniasis in Governador Valadares, Southeastern Brazil.” Scientific Reports 7, no. 1: 40804. 10.1038/srep40804. 28091623 PMC5238499

[cbdd70227-bib-0043] Vasconcelos, R. M. C. , F. C. Leite , J. A. Leite , S. Rodrigues Mascarenhas , L. C. Rodrigues , and M. R. Piuvezam . 2012. “Synthesis, Acute Toxicity and Anti‐Inflammatory Effect of Bornyl Salicylate, a Salicylic Acid Derivative.” Immunopharmacology and Immunotoxicology 34, no. 6: 1028–1038. 10.3109/08923973.2012.694891. 22712758

[cbdd70227-bib-0044] Vermeersch, M. , R. I. da Luz , K. Toté , J.‐P. Timmermans , P. Cos , and L. Maes . 2009. “In Vitro Susceptibilities of *Leishmania donovani* Promastigote and Amastigote Stages to Antileishmanial Reference Drugs: Practical Relevance of Stage‐Specific Differences.” Antimicrobial Agents and Chemotherapy 53, no. 9: 3855–3859. 10.1128/AAC.00548-09. 19546361 PMC2737839

[cbdd70227-bib-0045] Wadapurkar, R. M. , M. D. Shilpa , A. K. S. Katti , and M. B. Sulochana . 2018. “In Silico Drug Design for *Staphylococcus aureus* and Development of Host‐Pathogen Interaction Network.” Informatics in Medicine Unlocked 10: 58–70. 10.1016/j.imu.2017.11.002.

[cbdd70227-bib-0046] Wang, Y. , L. Long , Q. Yu , et al. 2023. “Discovery of Carbamate‐Based Salicylic Acid Derivatives as Novel Cholinesterase Inhibitor.” Journal of Molecular Structure 1276: 134804. 10.1016/j.molstruc.2022.134804.

[cbdd70227-bib-0047] Wodnicka, A. , E. Huzar , M. Krawczyk , and H. Kwiecień . 2017. “Synthesis and Antifungal Activity of New Salicylic Acid Derivatives.” Polish Journal of Chemical Technology 19, no. 1: 143–148. 10.1515/pjct-2017-0019.

[cbdd70227-bib-0048] World Health Organization . 2023. “Leishmaniasis.”

[cbdd70227-bib-0049] World Health Organization . 2024. “Neglected Tropical Diseases.”

[cbdd70227-bib-0050] Xu, C. , F. Cheng , L. Chen , et al. 2012. “In Silico Prediction of Chemical Ames Mutagenicity.” Journal of Chemical Information and Modeling 52, no. 11: 2840–2847. 10.1021/ci300400a. 23030379

