# Supplementary material for: Mitochondrial Dysfunction Induced by a Novel Salicylate‐Based 1,2,3‐Triazole Salt With Potent Antileishmanial Activity
Source: Chem Biol Drug Des. 2025 Dec 22;106(6):e70227. doi: 10.1111/cbdd.70227 (PMC12723204; doi:10.1111/cbdd.70227)

**Mitochondrial Dysfunction Induced by a Novel Salicylate-Based 1,2,3-Triazole Salt with Potent Antileishmanial Activity**

Bruno A. De Oliveira^1^; Ari Sérgio de Oliveira Lemos^2^; João Pedro Reis Costa Bastos^2^; Luciana Maria Ribeiro Antinarelli^2^; Ana Luiza Ribeiro de Freitas^2^, Raissa Guedes Mattosinhos Ribeiro^3^, Adolfo Firmino da Silva Neto^3^; Elaine Soares Coimbra^2^; Adilson David Da Silva ^1^*.

^1^Department of Chemistry, Institute of Exact Sciences, Federal University of Juiz de Fora, Juiz de Fora, Minas Gerais, CEP 36036-900, Brazil.

^2^Department of Parasitology, Microbiology and Immunology, Institute of Biological Sciences, Federal University of Juiz de Fora, Juiz de Fora, MG 36036-900, Brazil.

^3^Department of Veterinary Medicine, Faculty of Medicine, Federal University of Juiz de Fora, Juiz de Fora, MG 36036-900, Brazil.

* Corresponding author

E-mail address: david.silva@ufjf.br

**ADDITIONAL EXPERIMENTAL DETAILS**

**Fig. S1:** ^1^H NMR spectrum of compound **1** (500 MHz, CDCl_3_).

**Fig. S2:** ^13^C NMR spectrum of compound **1** (125 MHz, CDCl_3_).

**Fig. S3:** Infrared spectrum of compound **1**.

**Fig. S4:** ^1^H NMR spectrum of compound 2 (500 MHz, CDCl_3_).

**Fig. S5:** ^13^C NMR spectrum of compound **2** (125 MHz, CDCl_3_).

**Fig. S6:** Infrared spectrum of the compound **2**.

**Fig. S7:** ESI-MS spectrum of the compound **2**.


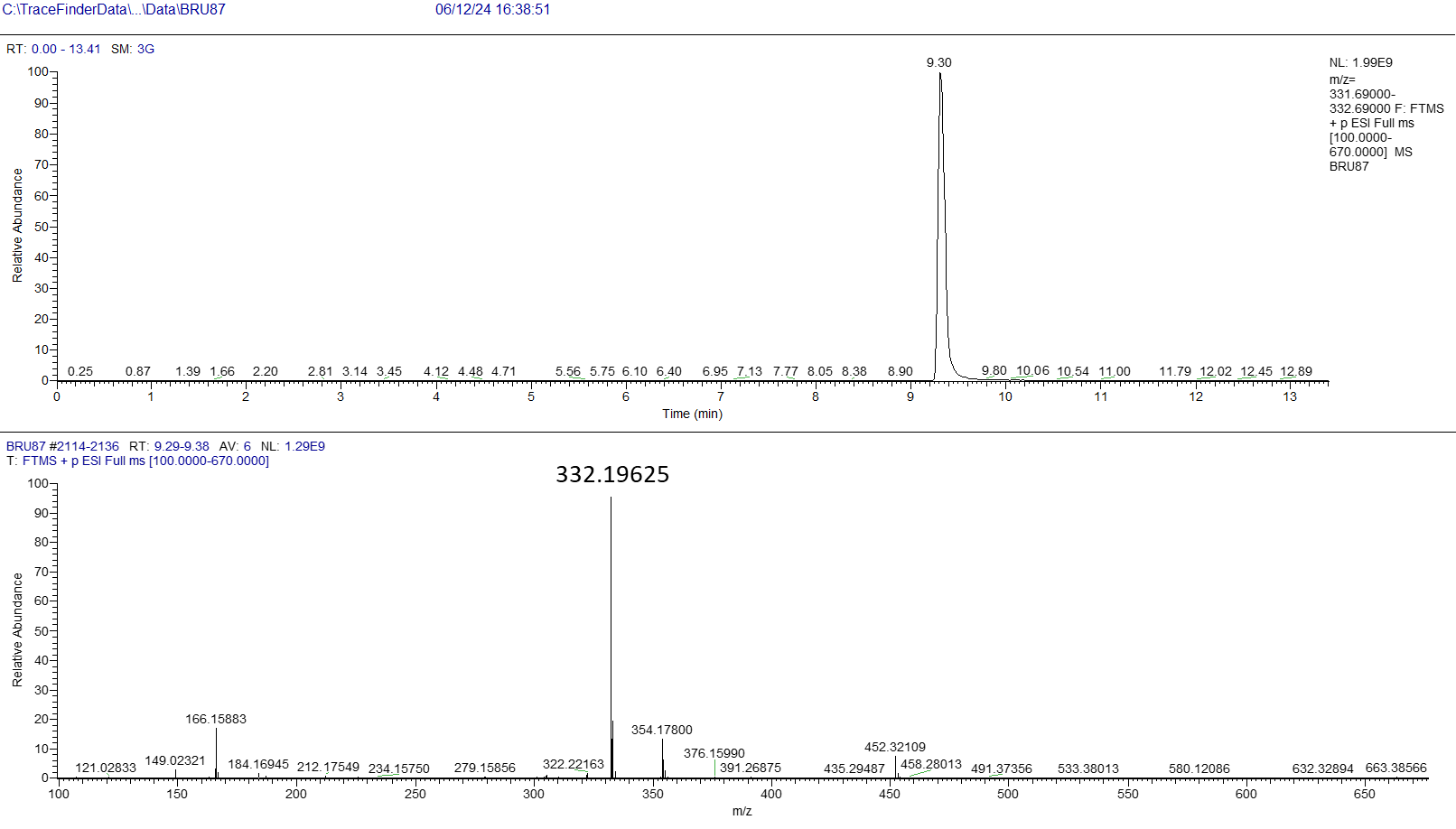


**Fig. S8:** ^1^H NMR spectrum of the compound **3** (500 MHz, CDCl_3_).

**Fig. S9:** ^13^C NMR spectrum of the compound **3** (125 MHz, CDCl_3_).

**Fig. S10:** Infrared spectrum of the compound **3**.

**Fig. S11:** ESI-MS spectrum of the compound **3**.


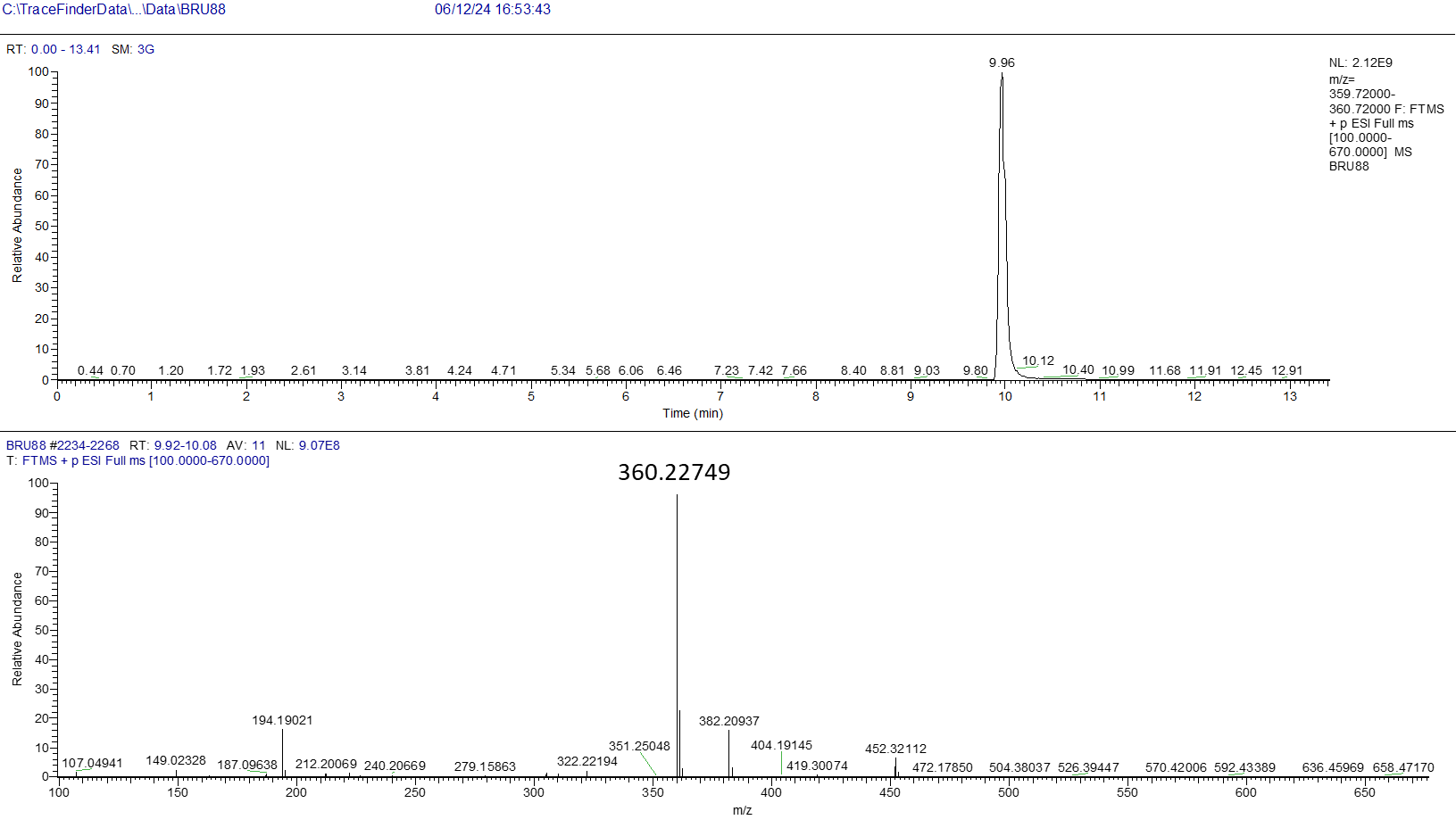


**Fig. S12:** ^1^H NMR spectrum of the compound **4** (500 MHz, CDCl_3_).

**Fig. S13:** ^13^C NMR spectrum of the compound **4** (125 MHz, CDCl_3_).

**Fig. S14:** Infrared spectrum of the compound **4**.

**Fig. S15:** ESI-MS spectrum of the compound **4**.


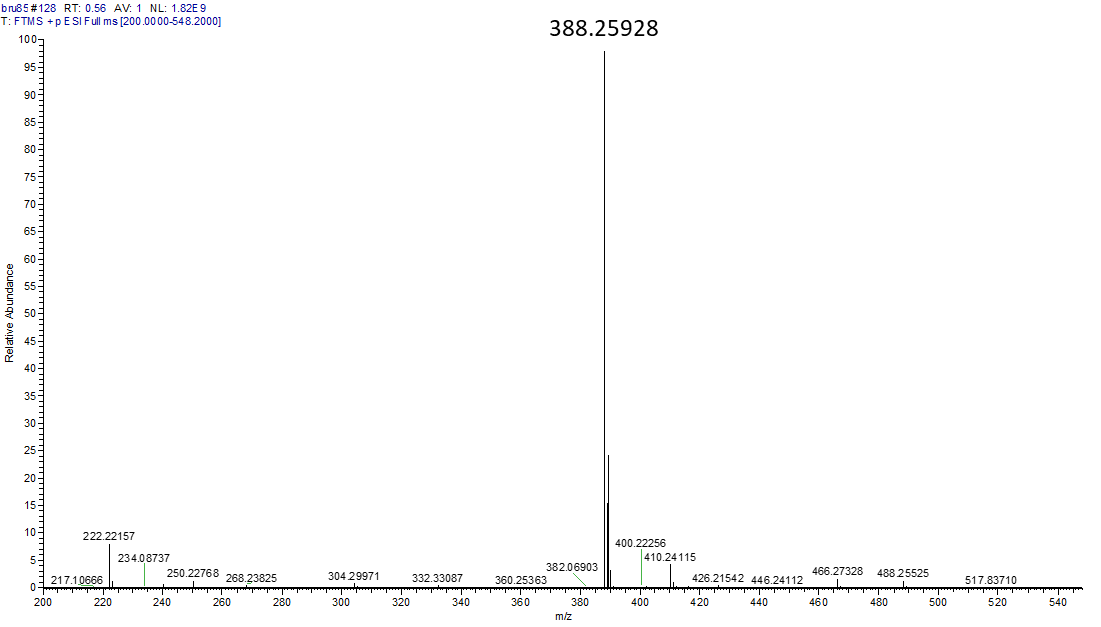


**Fig. S16:** ^1^H NMR spectrum of the compound **5** (500 MHz, CDCl_3_).

**Fig. S17:** ^13^C NMR spectrum of the compound **5** (125 MHz, CDCl_3_).

**Fig. S18:** Infrared spectrum of the compound **5**.

**Fig. S19:** ESI-MS spectrum of the compound **5**.


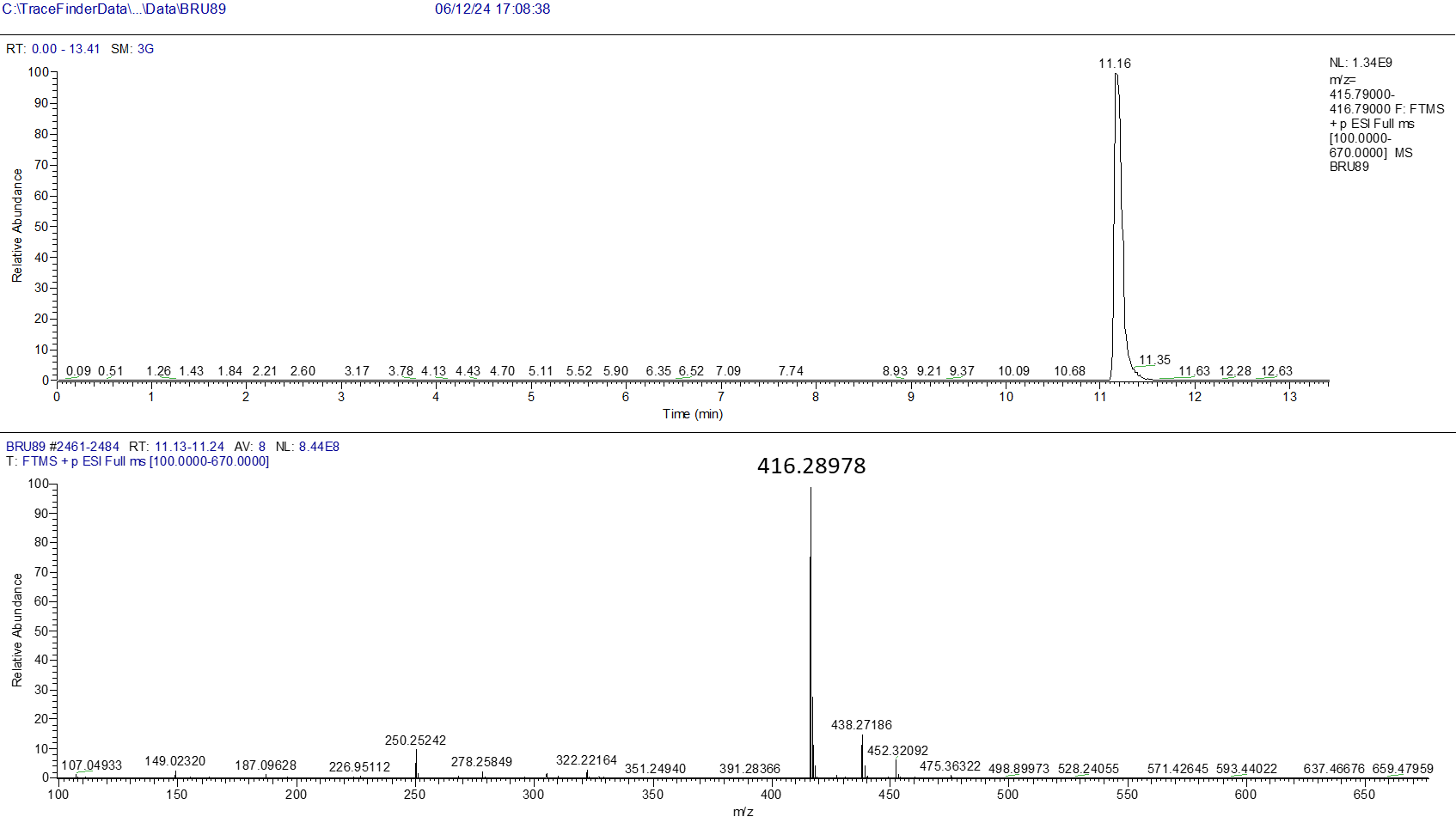


**Fig. S20:** ^1^H NMR spectrum of the compound **6** (500 MHz, CDCl_3_).

**Fig. S21:** ^13^C NMR spectrum of the compound **6** (125 MHz, CDCl_3_).

**Fig. S22**: Infrared spectrum of the compound **6**.

**Fig. S23:** ESI-MS spectrum of the compound **6**.


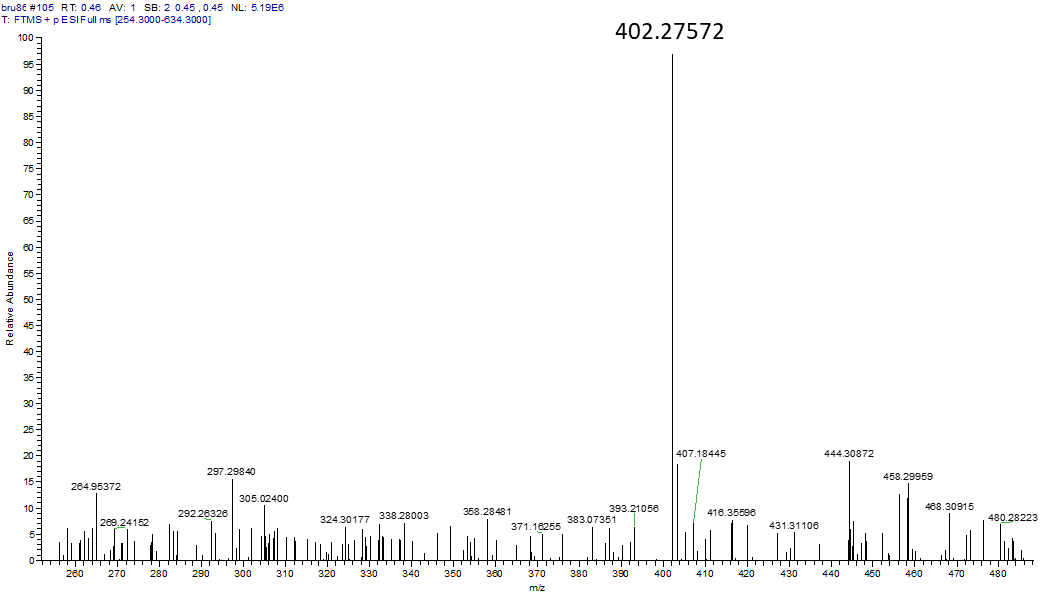


**Fig. S24:** ^1^H NMR spectrum of the compound **7** (500 MHz, CDCl_3_).

**Fig. S25:** ^13^C NMR spectrum of the compound **7** (125 MHz, CDCl_3_).

**Fig. S26:** Infrared spectrum of the compound **7**.

**Fig. S27:** ESI-MS spectrum of the compound **7**.


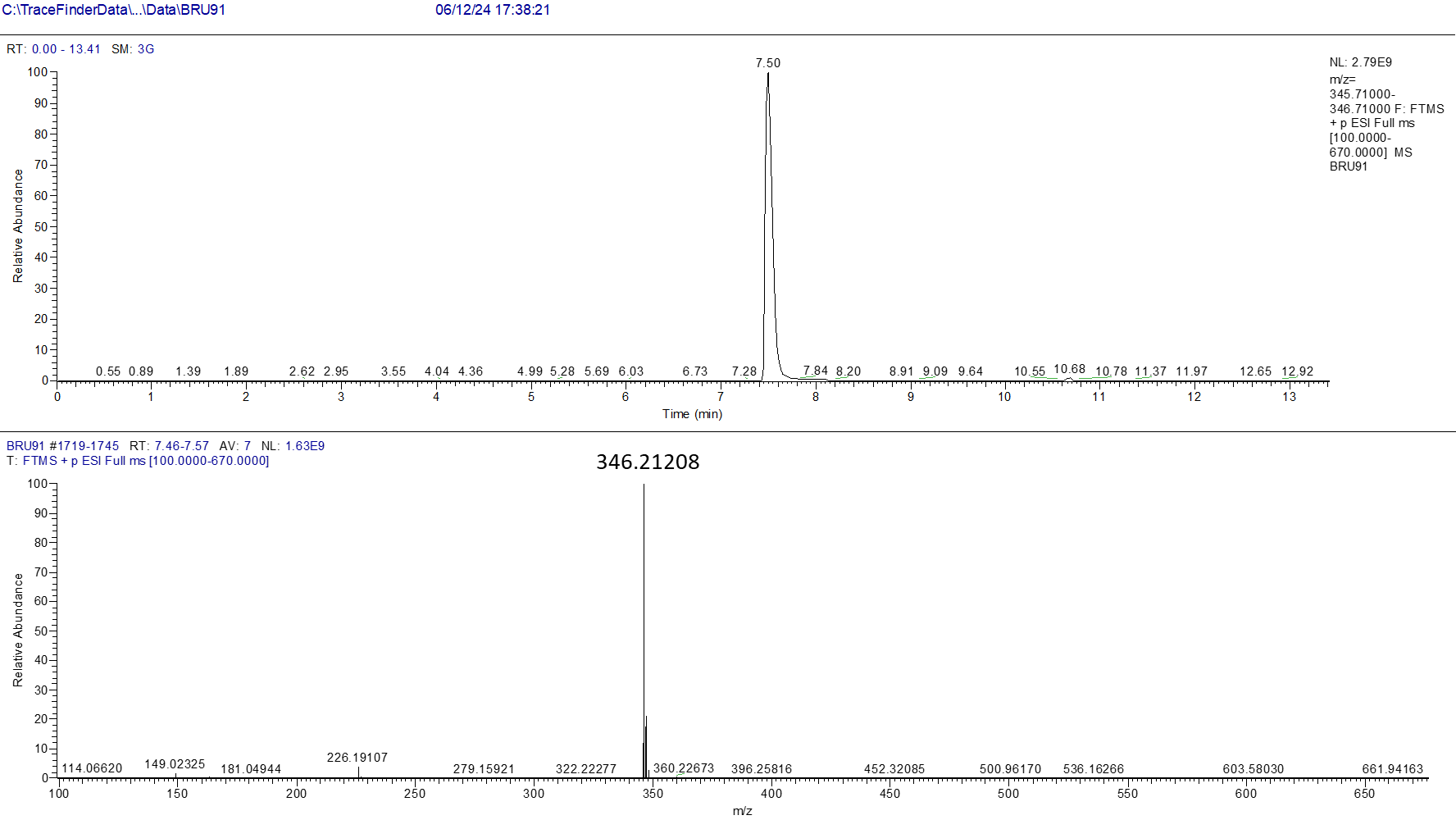


**Fig. S28:** ^1^H NMR spectrum of the compound **8** (500 MHz, CDCl_3_).

**Fig. S29:** ^13^C NMR spectrum of the compound **8** (125 MHz, CDCl_3_).

**Fig. S30:** Infrared spectrum of the compound **8**.

**Fig. S31:** ESI-MS spectrum of the compound **8**.


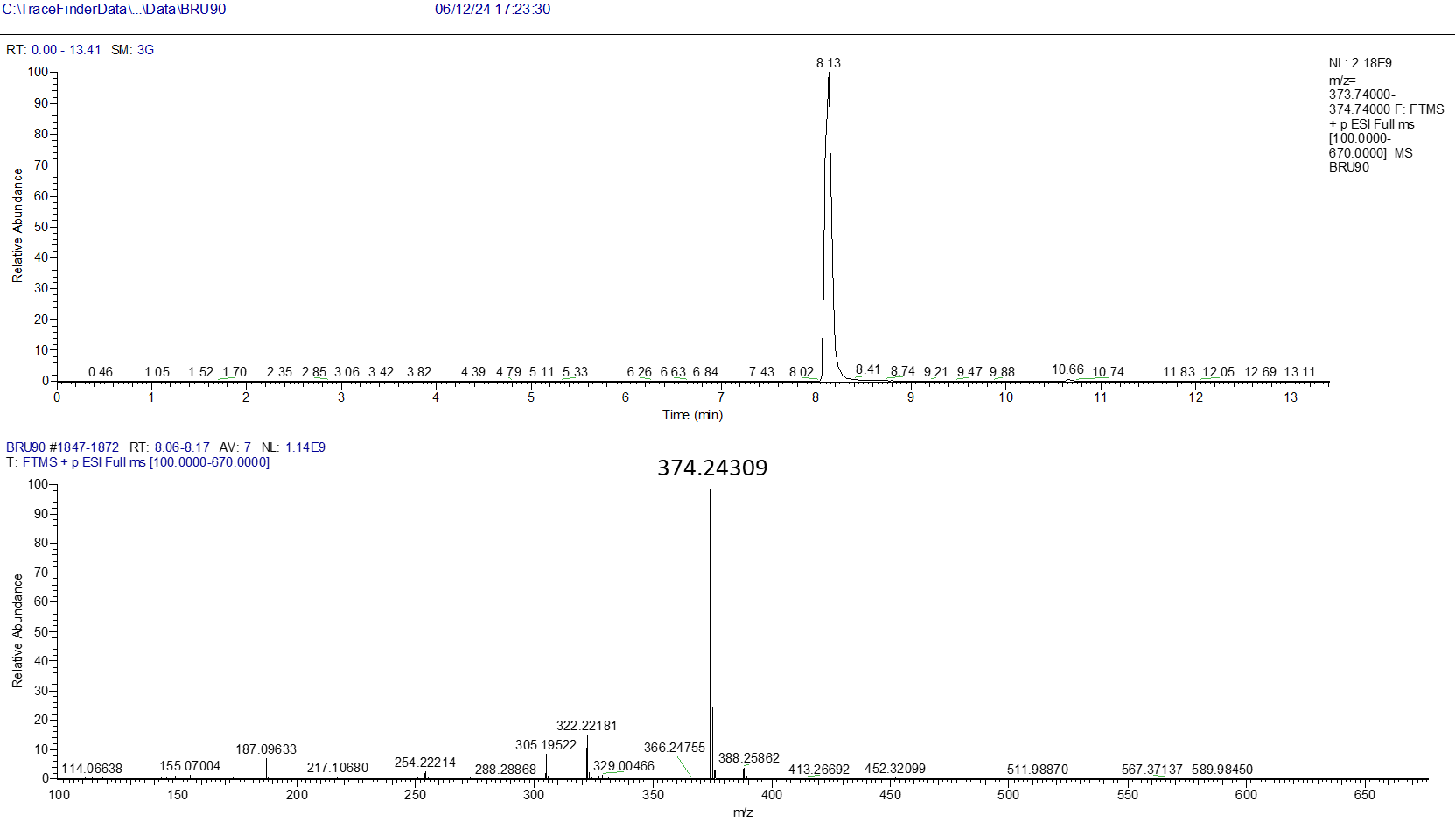


**Fig. S32:** ^1^H NMR spectrum of the compound **9** (500 MHz, CDCl_3_).

**Fig. S33:** ^13^C NMR spectrum of the compound **9** (125 MHz, CDCl_3_).

**Fig. S34:** Infrared spectrum of the compound **9**.

**Fig. S35:** ESI-MS spectrum of the compound **9**.


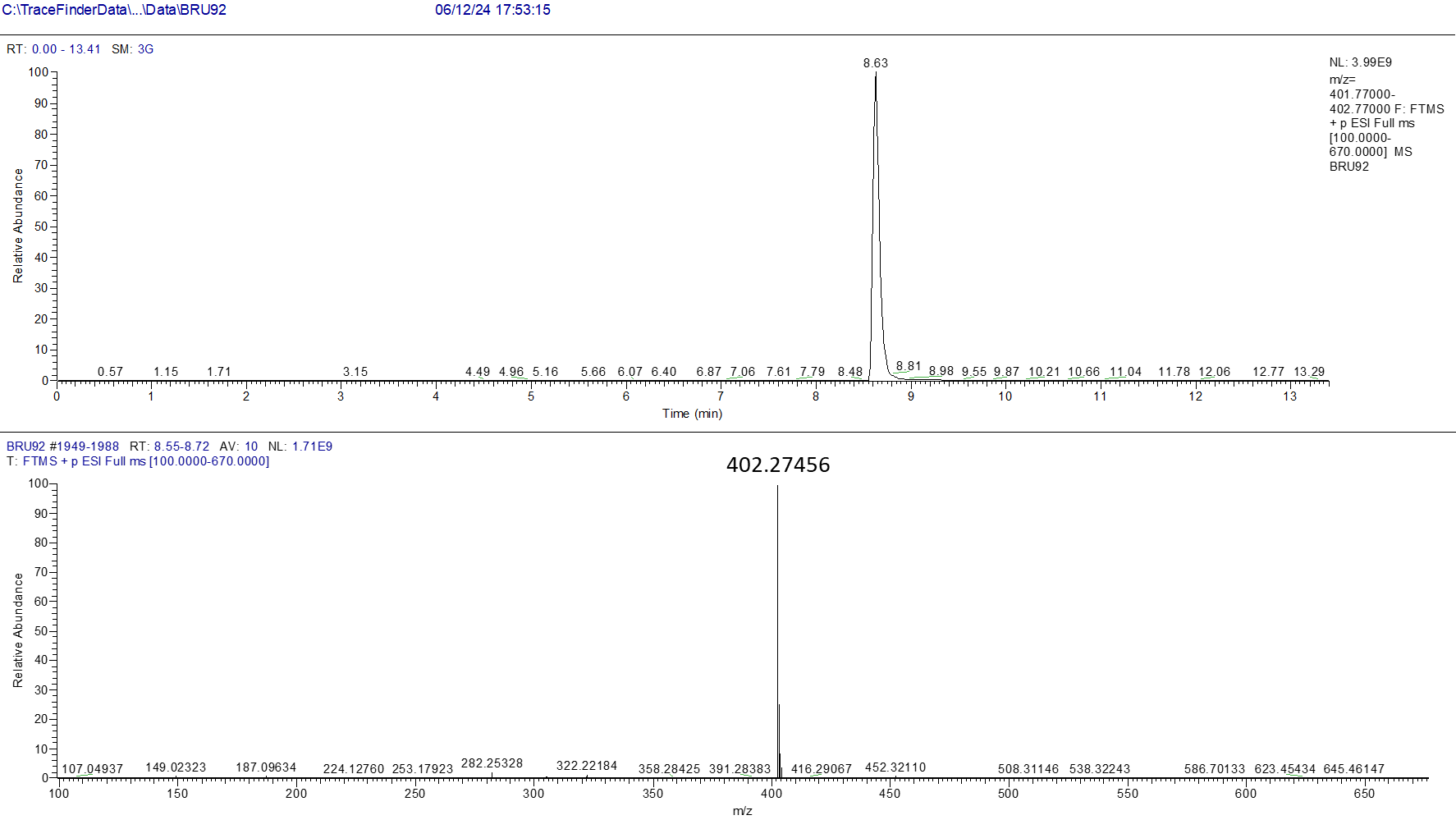


**Fig. S36:** ^1^H NMR spectrum of the compound **10** (500 MHz, CDCl_3_).

**Fig. S37:** ^13^C NMR spectrum of the compound **10** (125 MHz, CDCl_3_).

**Fig. S38:** Infrared spectrum of the compound **10**.

**Fig. S39:** ESI-MS spectrum of the compound **10**.


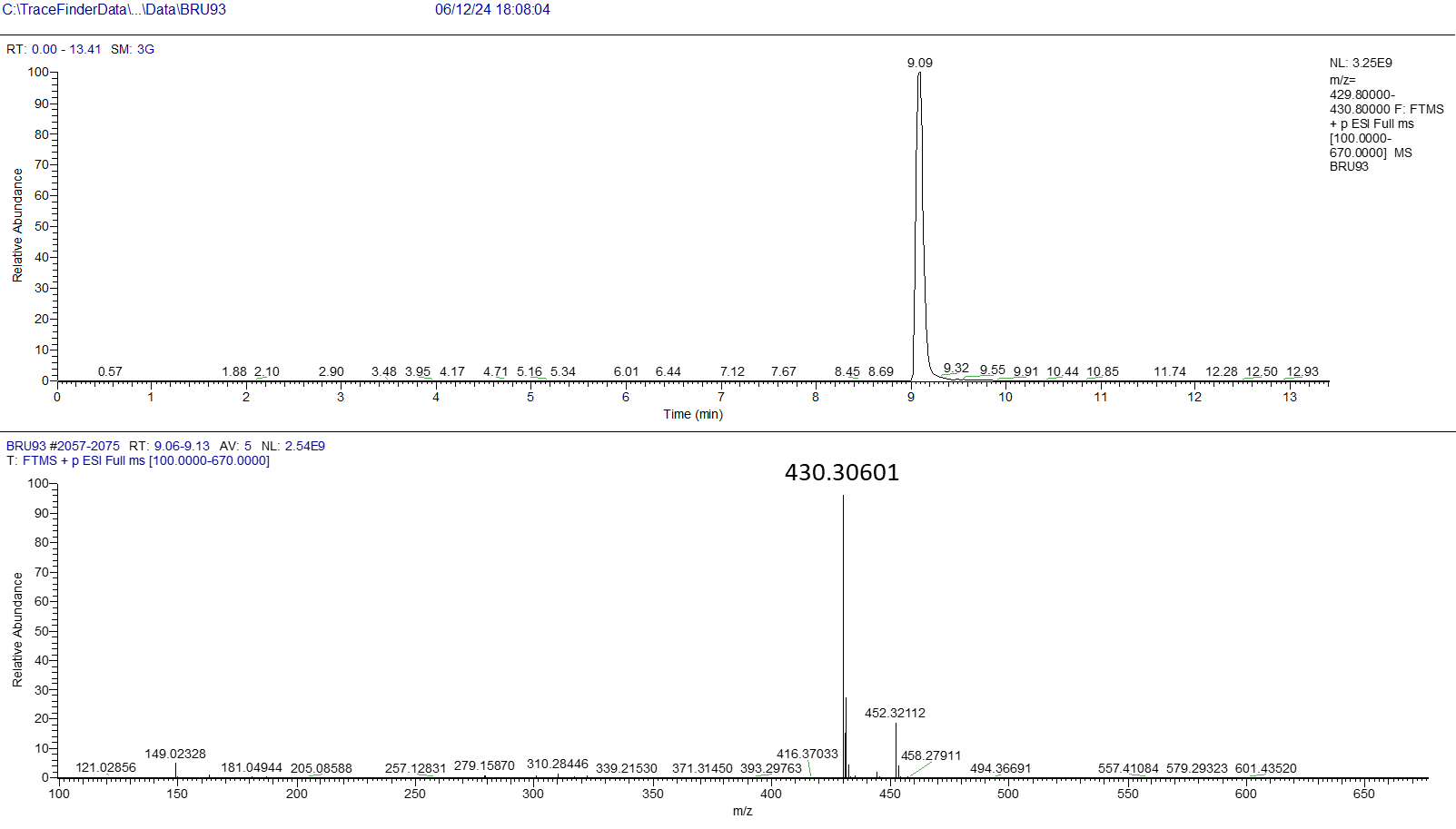


**Fig. S40:** ^1^H NMR spectrum of the compound **11** (500 MHz, CDCl_3_).

**Fig. S41:** ^13^C NMR spectrum of the compound **11** (125 MHz, CDCl_3_).

**Fig. S42:** Espectro de Infravermelho do Composto **11**.

**Fig. S43:** ESI-MS spectrum of the compound **11**.


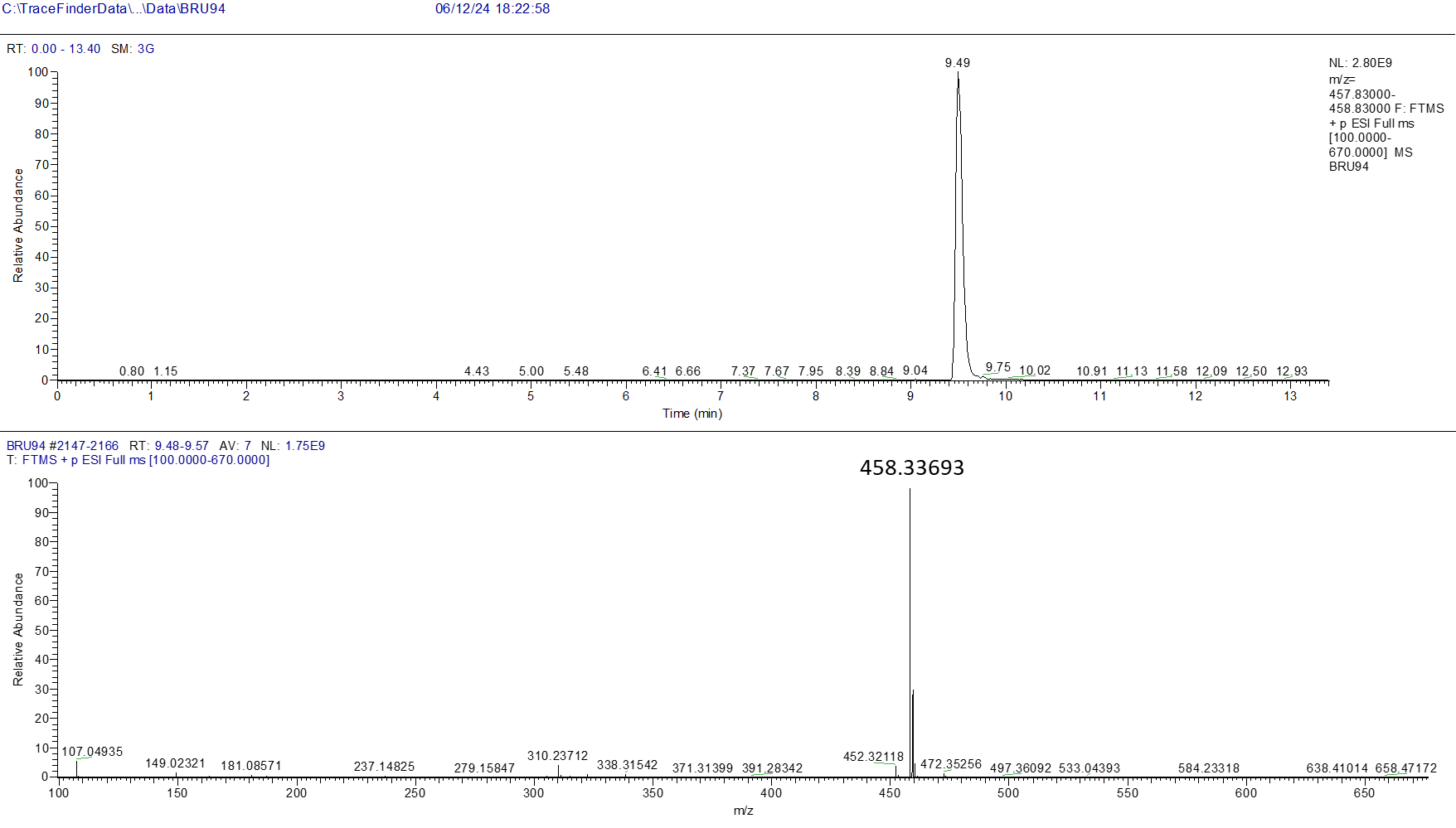

Supplement: Supplementary file 1 — Data S1: cbdd70227‐sup‐0001‐Figures.docx. [file CBDD-106-e70227-s001.docx]
